# Graphene inks for printing based on thermoresponsive ABC triblock terpolymer gels†

**DOI:** 10.1039/d5lp00071h

**Published:** 2025-06-02

**Authors:** Xu Liu, Bailin Feng, Stefano Tagliaferri, Anna P. Constantinou, Alexandra E. Porter, Cecilia Mattevi, Theoni K. Georgiou

**Affiliations:** a Department of Materials, Imperial College London, Royal School of Mines Exhibition Road SW7 2AZ London UK t.georgiou@imperial.ac.uk

## Abstract

Additive manufacturing has revolutionized the fabrication of complex 3D materials. Hydrogels are commonly used as “inks” in 3D printing and offer easy mixing and processing of many materials. Here, the synthesis and characterization of a new library of thermoresponsive ABC triblock copolymers based on oligo(ethylene glycol) methyl ether methacrylate (OEGMA, Molar Mass, MM = 300 g mol^−1^, A block), 2-phenylethyl methacrylate (PhEMA, B block) and di(ethylene glycol) methyl ether methacrylate (DEGMA, C block) is reported. Polymers of different comonomer compositions were fabricated and investigated in terms of their aqueous solution properties and their ability to form thermogels. The most promising polymer was then used to fabricate a graphene-containing ink, and graphene constructs were successfully printed and characterized in terms of the electrical conductivity properties.

## Abstract 2

In house synthesised ABC thermoresponsive methacrylate triblock terpolymers were used to create graphene based ink formulations for printing.

## Graphical abstract

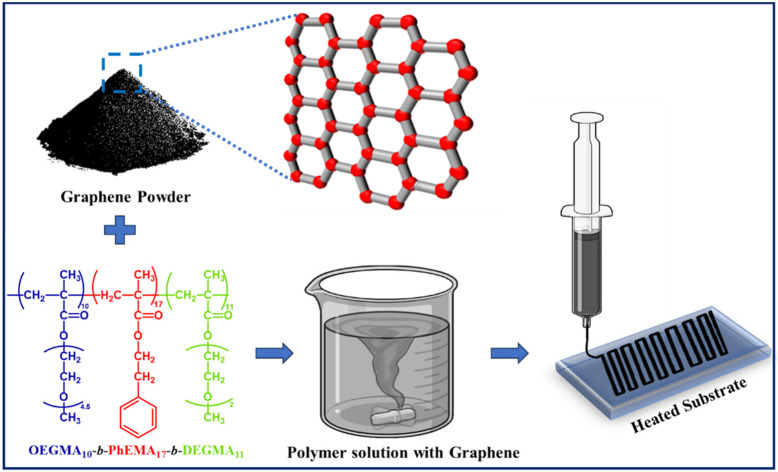

## Introduction

Additive manufacturing techniques, also called 3-Dimensional (3D) printing techniques have revolutionized fabricating methods over the last decades.^1–3^ In 3D printing, the designed structures/objects are manufactured layer-by-layer with computer-controlled translation stages following the data of the computer models. The digital assembly allows rapidly turning computer-aided designs into complex 3D prototypes without wasting excess materials, that is an advantage compared to conventional methods that require dies, molds, or lithographic masks.^1–3^ Consequently, due to this and the ability to rapidly fabricate products on demand, 3D printing technology has contributed to both industrial and academic research production. 3D printing has been used to fabricate constructs for energy, robotics, aerospace, and healthcare,^1–11^ while at the same time, it has strengthened the influence of polymers in our society.

Many different polymeric materials have been used for 3D printing including thermoplastics, thermosets, elastomers, functional polymers, polymer blends, composites and gels.^1–4,7–19^ Gels offer the advantage that they can be easily mixed with other ingredients, thus producing more intricate inks, and assisting the printing of conventionally non-printable materials. It is a prerequisite that all “inks” must exhibit non-Newtonian shear-thinning behavior that is a critical property to be considered in 3D extrusion printing.

Many different types of gels have been used in 3D printing but one of the most common components is Poloxamers, which are commercially available and have excellent rheological properties. Poloxamers are nonionic ABA triblock copolymers based on poly(ethylene glycol) (PEG, A block) and poly(propylene glycol) (PPG, B block) and have many trade names including Pluronic, Kolliphor and Synperonic. For example, Pluronic® F127 has been used to print graphene and copper inks to fabricate electrodes for electrochemical energy storage.^20^ Pluronic® F127 was incorporated in the inks to provide stabilization of the copper and graphene by preventing agglomeration. These inks were characterized by high storage modulus (in the range of 100 kPa), and stable structures were successfully printed.^20^

Pluronic® F127 solution was also used as a support gel for the printing of an end functionalized dimethacrylate Pluronic® F127, which was crosslinked while printing.^21^ Graphene oxide and multiwalled carbon nanotubes were incorporated into the ink and were successfully printed.

In some studies, Pluronic® F127 was used as the sacrificial material, *i.e.*, the component which is discarded after the final product is fabricated.^22,23^ For example a Pluronic® F127 gel was printed in pillar shape and the printed structure was immersed in agarose solution, which was gelled by heating to 35–45 °C, at which both samples were in the gel phase.^22^ Removal of the Pluronic® F127 construct was achieved by cooling the structure to 4 °C (liquid phase), thus a vascular structure was revealed.^22^ Similarly, in another study, Pluronic® F127 was used to create channels to mimic vascular structures.^23^ In this study, Pluronic® F127 gel was successfully printed in 30 layers. The same gel was printed on top of an alginate/gelatin block, and the resulting structure was covered by another alginate/gelatin block. After crosslinking the alginate component, the construct was then cooled down to 4 °C, so Pluronic® F127 gel was liquified and removed from the resulting vascular structure.^23^

In addition to mixing additives with the pre-synthesized copolymers, copolymers can also be synthesized directly in the presence of additives.^24^ For example, poly(glycerol monomethacrylate-*block*-hydroxypropyl methacrylate) (PGMA–PHPMA) graphene oxide gels have been fabricated using two distinct approaches. The first method included the incorporation of graphene oxide into pre-synthesized PGMA–PHPMA copolymers. In contrast, the second method involved the *in situ* synthesis of HPMA through reversible addition-fragmentation chain transfer (RAFT) polymerization, where PGMA functions as the macromolecular chain transfer agent, with graphene oxide being present throughout the process. Both resulting structures demonstrated good mechanical strength properties and were successfully printed.^24^

In-house synthesized block copolymers were also used for 3D printing. For example, an ABA triblock copolymer based on isopropyl glycidyl ether (A block) and PEG (B block) was successfully printed in 8 layers.^25^ A solution of an AB diblock copolymer based on 2-methyl-2-oxazoline and 2-*n*-propyl-2-oxazine was mixed with fibroblast cells, and the mixture was successfully printed at room temperature.^26^

Graphene, a single-layer atomic material,^27^ exhibits extraordinary mechanical strength^28^ and outstanding conductivity.^29^ These properties make it highly popular in conductive applications. However, its inherently non-polar nature results in poor dispersibility in aqueous solutions, which makes it difficult to be printed by 3D printer. Consequently, graphene is often functionalized or chemically modified prior to use,^30^ which in turn affects the conductivity of the final product. Therefore, we developed a series of polymers that contain an aromatic ring, which interact with graphene to function as inks for printing. Therefor here, to the best of our knowledge, we report the first study where ABC triblock copolymer solutions are used as inks for printing. The ABC architecture was chosen, as our group has previously proven than ABC triblock copolymers with the B block as the hydrophobic one show promise as thermoresponsive gels when compared to other terpolymer achitectures.^31–37^ The AB, BC architecture were chosen for comparison with the ABC architecture. In this study, a new library of thermoresponsive ABC triblock copolymers and two diblock copolymers (AB, CB) based on oligo(ethylene glycol) methyl ether methacrylate (OEGMA, Molar Mass, MM = 300 g mol^−1^, A block), 2-phenylethyl methacrylate (PhEMA, B block) and di(ethylene glycol) methyl ether methacrylate (DEGMA, C block) were synthesized *via* group transfer polymerization (GTP). The PhEMA monomer was chosen because it contains benzyl rings and it was hypothesized that it would assist with the stabilization of the graphene-based inks *via* π–π interactions, while DEGMA and OEGMA were chosen for their relatively low (∼30 °C) and high (∼75 °C) cloud point temperatures (*T*_cp_s), respectively,^38^ which were used to tailor the thermogelling abilities of the polymer. Polymers of varying compositions were fabricated because it is well established that the composition has a key role in the thermogelling properties,^19,31–37^ and their aqueous solution and thermoresponsive properties were studied. Furthermore, the most promising polymer was chosen to print a graphene containing ink.

## Experimental

### Materials

**Fig. 1:**
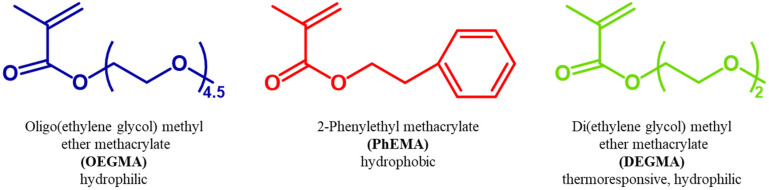
Chemical structures of the monomers OEGMA, PhEMA and DEGMA.

Di(ethylene glycol) methyl ether methacrylate (DEGMA, 95%), oligo(ethylene glycol) methyl ether methacrylate (OEGMA, MM = 300 g mol^−1^, 95%), 2-phenylethyl methacrylate (PhEMA, 99%), 1-methoxy-1-(trimethylsiloxy)-2-methylpropene (MTS, 95%), tetrahydrofuran (THF, ≥99.9%), 2,2-diphenyl-1-picrylhydrazyl (DPPH), calcium hydrate (CaH_2_, ≥90%), deuterated chloroform (CDCl_3_, 99.8%), acetone, *n*-hexane (>95%), tetrahydrofuran (THF, inhibitor-free, high-performance liquid chromatography (HPLC) grade, ≥99.9%), basic aluminum oxide and of poly(methyl methacrylate) (PMMA) standard samples (MM = 2000, 4000, 8000, 20 000, 50 000, 100 000 g mol^−1^) for the calibration of size exclusion chromatography (SEC) were bought from Sigma-Aldrich. The precursors of the catalyst, tetrabutylammonium bibenzoate (TBABB), were purchased from Acros Organics, U.K.: benzoic acid and tetrabutylammonium hydroxide (40% in water). Graphene (graphene nanoplatelets, 5 μm particle size) was purchased from Sigma Aldrich. The chemical structures of monomers used in this study are shown in Fig. 1.

### Monomers purification

The low MM monomers such as DEGMA and PhEMA were purified in a similar manner. Firstly, the monomer was based twice through basic alumina to remove any acidic impurities and collected in a round-bottom flask. Then, the free-radical inhibitor DPPH was added into the flask to prevent any unwanted polymerization. Consequently, the drying agent, CaH_2_, was added to the monomer and it was stirred for at least 4 hours under argon atmosphere. The monomers were then kept in the fridge and were distilled prior use. On the other hand, the OEGMA monomer, a high MM monomer, was firstly mixed with anhydrous THF (50 vol%/50 vol%) and then based through the basic alumina columns twice. No DPPH was added and only CaH_2_ to dry the monomer solution. This was left to stir for at least 4 hours under argon. The OEGMA monomer was not distilled and was directly syringed into the polymerization flask using a 0.45 μm PTFE filter to remove the solids, similar to the procedure established by Vamvakaki *et al.*^39^ MTS, the GTP initiator, was distilled directly under vacuum before polymerization. The catalyst TBABB was synthesized from benzoic acid and tetrabutylammonium hydroxide according to Dicker *et al.*^40^ All glassware were dried in the oven at 140 °C overnight prior use.

### ABC triblock terpolymer synthesis

**Fig. 2:**
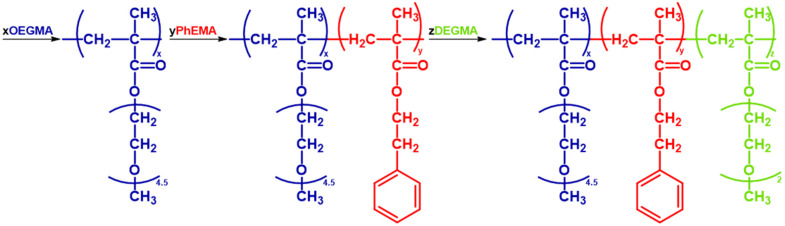
The sequential GTP synthesis of an OEGMA-*b*-PhEMA-*b*-DEGMA triblock copolymer. TBABB, THF and MTS represent the polymerization catalyst, solvent, and initiator respectively.

The amphiphilic ABC triblock terpolymers were synthesized *via* sequential GTP. A detailed example of a polymer synthesis follows. 53 mL of the polymerization solvent (THF) and 0.5 mL of the initiator (MTS, 0.43 g, 2.5 mmol) were syringed in a 250 mL round-bottom flask that was under argon and contained ∼10 mg of the TBBA catalyst. Then the monomers were added sequentially as shown in Fig. 2. First 15.6 mL of OEGMA solution (7.06 g, 0.02 mol) were syringed and an exotherm was observed. After ∼15 minutes, and when the temperature depleted 1 mL of the solution was withdrawn for Size Exclusion Chromatography (SEC) and Proton Nuclear Magnetic Resonance Spectroscopy (^1^H-NMR) analyses. Then, 5.2 mL of the second monomer, PhEMA (5.05 g, 0.03 mol) were injected into the polymerization flask. Again, after the temperature came back to room temperature a sample was taken for SEC and NMR. Finally, the last monomer was added. Specifically, 7.9 mL of DEGMA (8.07 g, 0.04 mol) were added and after 15 minutes a sample was again taken for SEC and NMR analyses. The reaction was terminated with 1 mL of methanol. All polymers were synthesized using a similar methodology and what was varied were the monomer ratios. The polymers were precipitated in cool *n*-hexane and were left to dry in a vacuum oven at room temperature for at least a week before use.

### Characterization in organic solvents

#### Size exclusion chromatography (SEC)

SEC was used to determine the number-average molar mass (*M*_n_), and dispersity (*Đ*) of all polymers and their precursors and to provide qualitative and quantitative information during polymer synthesis. An Agilent SECurity SEC system which was purchased from Agilent technologies UK Ltd (Shropshire) was used, equipped with a Mixed D column. The elution solvent was THF (95 vol%) + Et_3_N (5 vol%). The solvent was pumped with a 1 mL min^−1^ flow rate. The system was calibrated using six PMMA standard samples of varying molar masses (see Materials).

For the sample preparation ∼0.2 mL of the polymer solution (directly sampled from the polymerization flask) was added in a glass 2 mL vial. Then around 0.9 mL of solvent was added, and the sample was filters using a 0.45 μm PTFE filter before being analyzed.

#### Proton nuclear magnetic resonance spectroscopy (^1^H-NMR)

The chemical composition of polymers was determined *via*^1^H-NMR by using a 400 MHz Avance Bruker NMR spectrometer (Bruker, UK Ltd, Coventry, UK). The NMR solvent was deuterated chloroform (CDCl_3_). 10 mg of polymer dissolved in 650 μL of CDCl_3_ in a glass vial that was then transferred into an NMR tube for analysis.

### Characterization in aqueous solutions

#### Dynamic light scattering (DLS)

Zetasizer Nano ZSP (Malvern Instruments Ltd, Malvern, UK) was used to investigate the self-assembly behavior of polymers. 1 w/w% of polymer solution in DI water was fabricated for this analysis that were then filtered with 0.45 μm Nylon filters to remove big aggregates and impurities. Each sample was run for three time at 25 °C. Thus, the experimental hydrodynamic diameters (*d*_h_s) reported are the average of the maximum intensity values.

The *d*_h_s, as determined by DLS, are compared to the theoretical (calculated) diameters, which assume the formation of classical core–shell spherical micelles. The equation used for all ABC triblock terpolymers is the following: *d*_h_ (nm) = (DP_PhEMA_ + 2 × DP_OEGMA_ or DP_DEGMA_) × 0.254, depending on whether DP_OEGMA_ > DP_DEGMA_ or DP_OEGMA_ < DP_DEGMA_, respectively, where DP stands for degree of polymerization.^36,41^ For diblock copolymers, the equation is: *d*_h_ (nm) = (DP_hydrophobic block_ + 2 × DP_hydrophilic block_) × 0.254 (hydrophobic is always PhEMA while the hydrophilic block is either OEGMA or DEGMA).^42^ The DPs used in the calculations are experimentally determined, as resulted from ^1^H NMR spectroscopy, and the *M*_n_ values, as resulted from SEC analysis.

#### Visual tests

The aqueous polymer solutions were visually observed to investigate their thermoresponsive properties by heating up the vials in a water bath using an IKA RCT stirrer hot plate, equipped with an IKA ETS-D5 temperature controller. The polymer solutions in deionized water were investigated at a range of concentrations (1, 2, 5, 10, 15 and 20 w/w%) up to 80 °C. The samples were inspected for the following transitions: (i) runny solution (clear, slightly cloudy, and cloudy), (ii) viscous solution (transparent and cloudy), (iii) stable gel (transparent and cloudy), which is determined by the tube inversion method as the point that the sample that does not flow upon tube inversion, and (iv) two phases (gel syneresis, defined as gel disturbance due to internal stresses, and precipitation).

Visual tests of graphene/polymer mixtures in water were also performed. The graphene was kept constant at 10 w/w% while the polymer, P10: OEGMA_10_-*b*-PhEMA_17_-*b*-DEGMA_11_, concentration varied from 1 to 20 w/w%.

#### Turbidimetry using ultraviolet-visible (UV-vis) spectroscopy

The *T*_cp_s of 1 w/w% solutions were determined by an Agilent Cary UV-vis Compact Peltier UV-vis spectrometer. The polymer solutions were heated with a heating rate of 1 °C min^−1^ and data were collected every 1 °C at 550 nm. The *T*_cp_ was determined as the temperature at 50% transmittance.

#### Rheology test

The rheological properties of 15 w/w% and 20 w/w% polymer solutions in DI water were investigated *via* a TA Discovery HR-1 hybrid rheometer (TA Instruments UK, Waters Ltd, Hertfordshire, UK), equipped with a 40 mm parallel Peltier steel plate and a solvent trap. The rheological and the shear-thinning properties of the graphene inks (P10 10 w/w% + 10 w/w% graphene in DI water) were also investigated. The variations of rheological properties such as shear storage modulus (*G*′) and shear loss modulus (*G*′′) were determined while the temperature was increased from 20 °C to 60 °C. The measurements were taken using a 1 °C min^−1^ temperature ramp rate, a 1% strain, a 1 rad per s angular frequency, and a plate gap of 500 μm.^43^ For the shear-thinning property test, the temperature was maintained at 40 °C, while the shear rate varied from 0.01 s^−1^ to 100 s^−1^.

### Printing polymer/graphene aqueous solutions

The graphene inks (P10 10 w/w% + 10 w/w% graphene) were sonicated for 5 minutes and loaded into 3 mL polypropylene syringes with blunt nozzles (400 μm inner diameter) for Direct Ink Writing. The Direct Ink Writing of the inks was performed using a customized 3D printer (3D Inks, LLC), consisting of a Cartesian coordinate robot with a nominal precision of 1 μm. The syringes were mounted onto the three-axis stage of the 3D printer and connected to a displacement-controlled plunger, which provided a feed rate of 6 mm s^−1^. RoboCAD 5 was used to design the CAD model and convert it into G-code. P10: OEGMA_10_-*b*-PhEMA_17_-*b*-DEGMA_11_/graphene was printed onto glass substrates heated to 40 °C using a hotplate.

### Conductivity measurements

The electrical conductivity of the printed structure was tested utilizing a four-electrode measurement. Conductive silver paint (RSPRO conductive paint) was used to create four equally spaced contacts, each spaced 1.2 cm apart, on a printed line. A Gamry interface 1000 galvanostat was used to obtain the *I*–*V* curve of the printed structure through a 4-probe measurement. The electrical conductance *G* was determined by fitting the obtained *I*–*V* curve with a linear fitting. Subsequently, the electrical conductivity *σ* was calculated from the conductance *G*, the equation of which is:
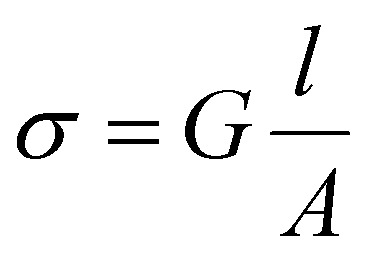
where *l* is the distance between the contacts and *A* is the cross-sectional area of the printed line.

## Results and discussion

### Polymer synthesis

All polymers were fabricated *via* GTP, a living polymerization method, that specializes on methacrylate polymers.^44–46^ Two diblock copolymers (AB, BC) and a series of ABC triblock copolymers based on OEGMA, PhEMA and DEGMA of varying composition were fabricated. PhEMA was chosen to be the B block of the ABC triblock copolymers, because our group has established that this architecture has better sol–gel thermogelling properties in triblock copolymers based on different monomers.^31,33–36,47^

### Molar mass and composition

**Fig. 3:**
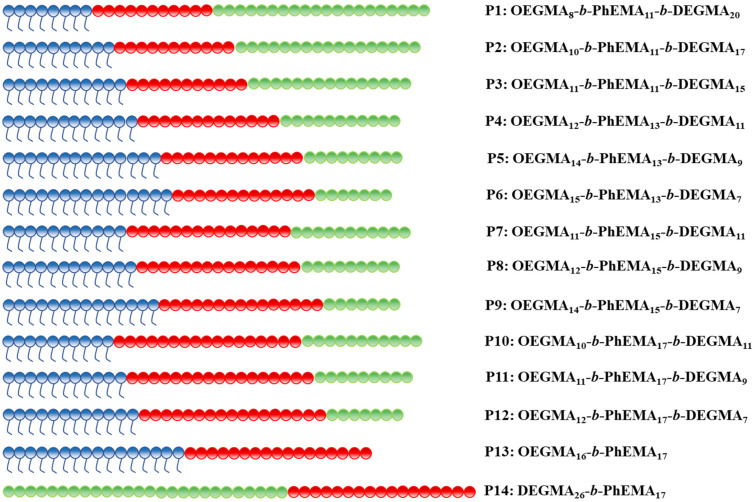
Schematic illustration of the synthesized OEGMA-*b*-PhEMA-*b*-DEGMA copolymers. OEGMA, PhEMA and DEGMA units are represented in blue, red, and green, respectively.

The target MM of all copolymers was 8000 g mol^−1^, based on previous studies indicating that copolymers with MMs in the range of 7000 to 10 000 g mol^−1^ exhibit optimal thermoresponsive behavior, including a clear sol–gel transition and good mechanical properties.^41,48^ In this study, four groups with four different compositions of PhEMA were investigated: 25 w/w% (P1–P3), 30 w/w% (P4–P6), 35 w/w% (P7–P9), and 40 w/w% (P10–P14). The ratio of the OEGMA and DEGMA units was varied to tailor the thermoresponsive property of triblock polymers OEGMA-*b*-PhEMA-*b*-DEGMA in each group. The structures of triblock polymers and diblock polymers are schematically illustrated in Fig. 3. OEGMA, PhEMA and DEGMA units are represented in blue, red, and green, respectively.

**Fig. 4:**
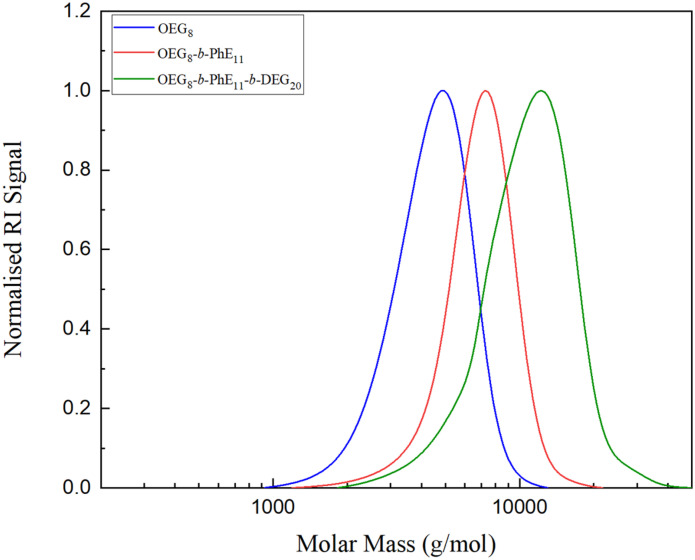
The SEC traces of P1: OEGMA_8_-*b*-PhEMA_11_-*b*-DEGMA_20_ and its precursors OEGMA_8_ and OEGMA_8_-*b*-PhEMA_11_ are shown in blue, red and green, respectively.

The MM characteristics and dispersity (*Đ*) of diblock and triblock polymers and their corresponding precursors were determined by SEC. The SEC traces of the precursors of P1 and the final synthesized polymer, OEGMA_8_-*b*-PhEMA_11_-*b*-DEGMA_20_, are shown in Fig. 4. The precursors, OEGMA_8_ and OEGMA_8_-*b*-PhEMA_11_ are denoted with blue and red curves respectively, while the ABC triblock copolymer OEGMA_8_-*b*-PhEMA_11_-*b*-DEGMA_20_ is in green. It can be seen from the SEC traces that the MM increased at each polymerization step and that no other peaks or shoulders are presented confirming a successful sequential GTP. This was similar for all synthesized copolymers (see Fig. S1 in ESI†).

The theoretical polymer structure, theoretical MM, experimental determined number average MM (*M*_n_), dispersity (*Đ*), theoretical and experimental composition of all synthesized polymers are listed in Table 1. To maintain clarity in the main text, detailed data for the polymer precursors are provided separately in Table S1 in the ESI.† The *M*_n_s and *Đ*s were obtained from SEC measurement, while the experimental compositions were determined by NMR. The data confirms the successful synthesis of all polymers, each exhibiting a narrow MM distribution (MMD) with *Đ* values ranging from 1.10 to 1.20.

**Table 1:** Theoretical polymer structure, target, and experimental molecular mass (*M*_n_), dispersity (*Đ*), theoretical and experimental composition of polymers

No.	Theoretical polymer structure^a^	Target MM (g mol^−1^)	*M*_n _ b (g mol^−1^)	*Đ* b	w/w% OEG-*b*-PhE-*b*-DEG	
					Theoretical	^1^H NMR
P1	OEG_8_-*b*-PhE_11_-*b*-DEG_20_	8200	9700	1.20	30-25-45	31-24-45
P2	OEG_10_-*b*-PhE_11_-*b*-DEG_17_	8200	9870	1.17	35-25-40	38-25-37
P3	OEG_11_-*b*-PhE_11_-*b*-DEG_15_	8200	10 100	1.15	40-25-35	43-24-33
P4	OEG_12_-*b*-PhE_13_-*b*-DEG_11_	8200	9550	1.12	45-30-25	47-29-24
P5	OEG_14_-*b*-PhE_13_-*b*-DEG_9_	8200	9550	1.13	50-30-20	52-30-18
P6	OEG_15_-*b*-PhE_13_-*b*-DEG_7_	8200	9810	1.11	55-30-15	59-28-13
P7	OEG_11_-*b*-PhE_15_-*b*-DEG_11_	8200	10 500	1.14	40-35-25	43-33-24
P8	OEG_12_-*b*-PhE_15_-*b*-DEG_9_	8200	11 300	1.13	45-35-20	47-33-20
P9	OEG_14_-*b*-PhE_15_-*b*-DEG_7_	8200	8490	1.13	50-35-15	51-34-15
P10	OEG_10_-*b*-PhE_17_-*b*-DEG_11_	8200	8200	1.16	35-40-25	36-39-25
P11	OEG_11_-*b*-PhE_17_-*b*-DEG_9_	8200	8600	1.15	40-40-20	42-39-19
P12	OEG_12_-*b*-PhE_17_-*b*-DEG_7_	8200	9000	1.14	45-40-15	47-38-15
P13	OEG_16_-*b*-PhE_17_	8200	8300	1.17	60-40-0	61-39-0
P14	DEG_26_-*b*-PhE_17_	8200	10 400	1.17	0-40-60	0-40-60

a OEG, PhE and DEG are further abbreviations of oligo(ethylene glycol) methyl ether methacrylate, 2-phenylethyl methacrylate and di(ethylene glycol) methyl ether methacrylate, respectively.

b Number-average molar mass (*M*_n_) and dispersity (*Đ*) were determined by SEC. The SEC was calibrated by using poly(methyl methacrylate) (PMMA) standard samples.

As shown in Table 1, the *Đ* value of each polymer ranges from 1.10 to 1.20, which is satisfactory low for polymers obtained *via* GTP when a OEG(PEG)-based methacrylate macromonomer was used in the polymerisation.^31,33–36,39,47–50^ This is because the OEGMA monomer is a macromonomer and has its own MMD and also it cannot be purified as well as the other monomers, resulting to broader MMD.

When comparing the theoretical MM with the experimental number average MM, *M*_n_ values in Table 1, it can be concluded that some deactivation was presented for all polymerizations, thus resulting to higher MM than theoretically predicted. This was expected and observed before in other GTP studies when using the OEGMA monomer.^31,33–36,39,49,50^ Specifically, the *M*_n_s for all polymers ranged from 8 to 11 kg mol^−1^.

**Fig. 5:**
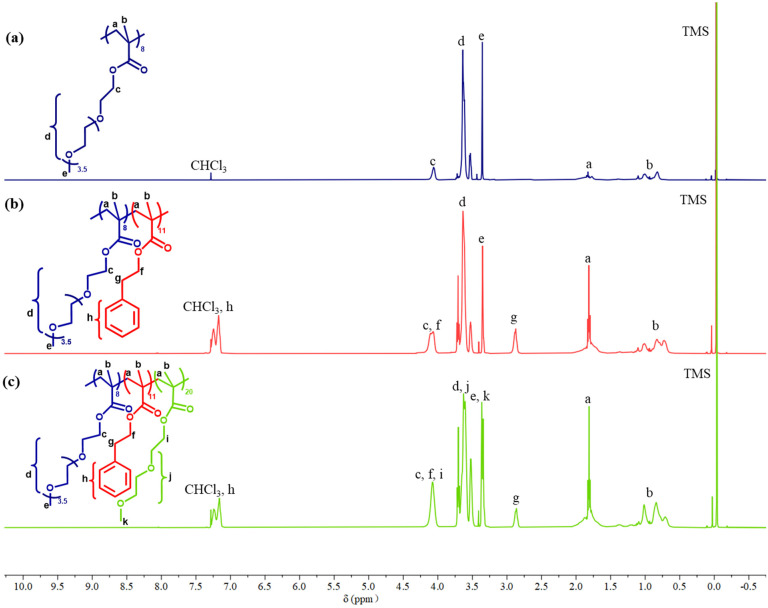
^1^H NMR spectra of (a) homopolymer OEGMA_8_, (b) diblock polymer OEGMA_8_-*b*-PhEMA_11_, and (c) ABC triblock terpolymer OEGMA_8_-*b*-PhEMA_11_-*b*-DEGMA_20_. The chemical structures of the corresponding polymers are also shown and the different ^1^H are labelled in the spectra.

The experimental compositions determined by ^1^H NMR, along with theoretical compositions, are also provided in Table 1 for the final polymers and the same information for both the precursors to the polymers and the copolymers is provide in Table S1.†Fig. 5 shows the ^1^H NMR spectra of P1: OEGMA_8_-*b*-PhEMA_11_-*b*-DEGMA_20_ and its linear precursors. It can be observed there are many overlapping peaks between OEGMA and DEGMA because of similar chemical structures, while the PhEMA has one distinct peak at 2.8–3.0 ppm in the NMR spectrum. Therefore, the ratio of OEGMA/PhEMA/DEGMA could not be directly calculated *via* the NMR spectrum of triblock polymer Fig. 5(c). Therefore, the NMR spectrum of the precursors (diblock polymer) Fig. 5(b) was used to obtain the ratio of OEGMA/PhEMA and the ratio of (OEGMA + DEGMA)/PhEMA was calculated *via* the NMR spectrum of the triblock polymer Fig. 5(c). Then, the DEGMA/PhEMA was calculated by using the ratio of (OEGMA + DEGMA)/PhEMA minus the ratio of OEGMA/PhEMA. Thus, the ratio of OEGMA/PhEMA/DEGMA was obtained. All the determined ratios are presented in Table 1 and Table S1,† and it can be concluded that the experimental compositions are sufficiently close to the theoretical compositions, thus confirming a successful synthesis.

### Aqueous properties

The aqueous and thermoresponsive properties of the ABC triblock polymers (1 w/w%) in DI water were investigated by DLS and UV-Vis and the results are presented in Table 2. All polymers are soluble in water, except for P1 and P14. This is due to the high hydrophobicity of these two copolymers.

**Table 2:** Theoretical polymer structure, PhEMA composition, theoretical and experimental hydrodynamic diameter, polydispersity index (PDI) and cloud point temperatures (*T*_cp_s) of 1 w/w% polymer solutions in DI water

No.	Theoretical polymer structure^a^	w/w% PhEMA	Hydrodynamic diameter (*d*_h_, nm)			Cloud point Temperature (*T*_cp_, °C)
			Theor.^b^	Experim.^c^	PDI	UV-vis (± 1)
P1	OEG_8_-*b*-PhE_11_-*b*-DEG_20_	25	—	—d	—d	—d
P2	OEG_10_-*b*-PhE_11_-*b*-DEG_17_	25	11.9	13.5	0.053	48
P3	OEG_11_-*b*-PhE_11_-*b*-DEG_15_	25	12.7	13.5	0.040	54
P4	OEG_12_-*b*-PhE_13_-*b*-DEG_11_	30	11.9	15.7	0.056	56
P5	OEG_14_-*b*-PhE_13_-*b*-DEG_9_	30	13.0	18.2	0.065	55
P6	OEG_15_-*b*-PhE_13_-*b*-DEG_7_	30	13.7	18.2	0.123	57
P7	OEG_11_-*b*-PhE_15_-*b*-DEG_11_	35	12.7	21.0	0.059	56
P8	OEG_12_-*b*-PhE_15_-*b*-DEG_9_	35	13.5	24.4	0.104	59
P9	OEG_14_-*b*-PhE_15_-*b*-DEG_7_	35	12.4	24.4	0.112	52
P10	OEG_10_-*b*-PhE_17_-*b*-DEG_11_	40	9.8	18.2	0.054	45
P11	OEG_11_-*b*-PhE_17_-*b*-DEG_9_	40	10.6	18.2	0.039	52
P12	OEG_12_-*b*-PhE_17_-*b*-DEG_7_	40	11.7	21.0	0.037	49
P13	OEG_16_-*b*-PhE_17_	40	12.9	24.4	0.077	55
P14	DEG_26_-*b*-PhE_17_^d^	40	—	—d	—d	—d

a OEG, PhE and DEG are further abbreviations of oligo(ethylene glycol) methyl ether methacrylate, 2-phenylethyl methacrylate and di(ethylene glycol) methyl ether methacrylate respectively.

b The calculation of theoretical diameter is based on the polymer structure of this polymer series and experimental degree of polymerization (DP). For ABC triblock polymers, two equations are applied: *d*_h_ (nm) = (DP_B_ + 2 × DP_A_) × 0.254 or *d*_h_ (nm) = (DP_B_ + 2 × DP_C_) × 0.254. It depends on if DP_A_ > DP_C_ or DP_C_ > DP_A_.^36,41^ For AB diblock polymers, the equation is: *d*_h_ (nm) = (DP_B_ + 2 × DP_A_) × 0.254.^42^ For all polymers in this series, A, B and C represent OEGMA, PhEMA and DEGMA respectively.

c The experimental diameters shown are the average values of the values of peaks by intensity in the DLS curves.

d P1 (OEG_8_-*b*-PhE_11_-*b*-DEG_20_) and P14 (DEG_26_-*b*-PhE_17_) are insoluble at 1 w/w% in DI water.

### Hydrodynamic diameters

The hydrodynamic diameters were measured by DLS. To compare with experimental data, theoretical hydrodynamic diameters were calculated based on the polymer structures and experimentally determined DP, using the equation described in the Characterization in aqueous solution section. These calculations are based on the assumption that the copolymers self-assemble into spherical micelles with a core-shell structure in aqueous solution.

**Fig. 6:**
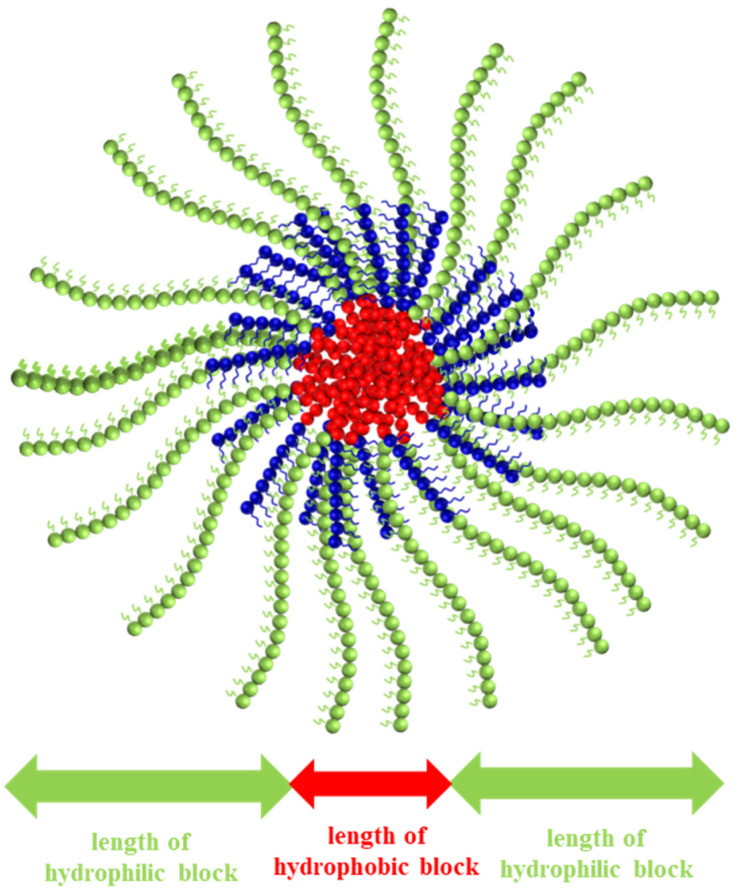
A schematic illustration of a spherical micelle with the core-shell structure of P1 OEGMA_8_-*b*-PhEMA_11_-*b*-DEGMA_20_. The units of OEGMA, PhEMA and DEGMA are represented in blue, red, and green spheres respectively.

A spherical micelle with the core-shell structure of P1: OEGMA_8_-*b*-PhEMA_11_-*b*-DEGMA_20_ is schematically illustrated in Fig. 6. In this illustration, the units of OEGMA, PhEMA and DEGMA are represented in blue, red and green spheres respectively. The core of the spherical micelle consists of PhEMA units, and the shell of the spherical micelle is made from OEGMA and DEGMA units. All other copolymers self-assemble in a similar way to form spherical micelles with the core-shell structure as shown in Fig. 6.

It can be seen from Fig. 6 that the size of the micelle depends on the length of hydrophilic part and hydrophobic part. The hydrodynamic diameter (*d*_h_) of the copolymers synthesized in this paper varied from 13.5–24.4 nm. The experimental diameters are slightly higher than the theoretical diameters in all samples as can be observed in Table 2 (DLS histograms are provided in Fig. S3 in the ESI†). Similar results have been reported in our previous study.^30,31,36,39,51^ This is because the theoretical calculations do not consider the long OEG side chain of OEGMA300 and assume complete overlap of the hydrophobic part (PhEMA block) of the polymer.^44^ In addition, the PDI values range from 0.040 to 0.123 in Table 2, thus are satisfactory low and no other peaks were detected by intensity. This suggests that the size of the self-assembled particles in polymer solutions is uniform.

### Cloud points temperatures

The *T*_cp_s by turbidimetry of 1 w/w% polymer solutions in DI water are presented in Table 2 and the transmittance *versus* temperature graphs are provided in Fig. S4 in the ESI.† The *T*_cp_s range from 45 to 59 °C and different trends are observed as the hydrophilic OEGMA component is increased. Specifically, for the 25 w/w%, 30 w/w% and 40 w/w% of PhEMA, the *T*_cp_ decreases as the hydrophilic OEGMA component decreases, as it was observed before.^33,35,37,48,52,53^ P10 (OEG_10_-*b*-PhE_17_-*b*-DEG_11_) exhibits the lowest *T*_cp_ as expected, which is 45 °C. This is because P10 is the most hydrophobic polymer among the 13 copolymers studied, with 40 w/w% PhEMA content. However, for the 35 w/w% PhEMA copolymers the opposite trend is observed, *i.e.*, the *T*_cp_s decrease with increasing the hydrophilic OEGMA content. Even more surprising, at the intermediate hydrophobic content of 35 w/w% a maximum is observed as the hydrophilic content is increased. These opposite trends could be explained by the fact that all the polymers are in a self-assembled structure and what could have key role in the *T*_cp_ is the corona of the micelle that is consisted mostly by the OEGMA content. Thus, it is believed that the size of the corona will have the biggest influence and as this is increased, the *T*_cp_ will increase, as the polymer will be able to stabilize themselves in solution better (for higher temperatures). Therefore, P8 (OEG_12_-*b*-PhE_15_-*b*-DEG_9_) has the highest *T*_cp_ because of the biggest micelle size. In addition, it should also be pointed out that this the theoretical DP and MM differences will also affect the *T*_cp_ as it has established that the MM also has a significant effect on the *T*_cp_.^37,48,51,54–56^

### Gelation points by visual tests

**Fig. 7:**
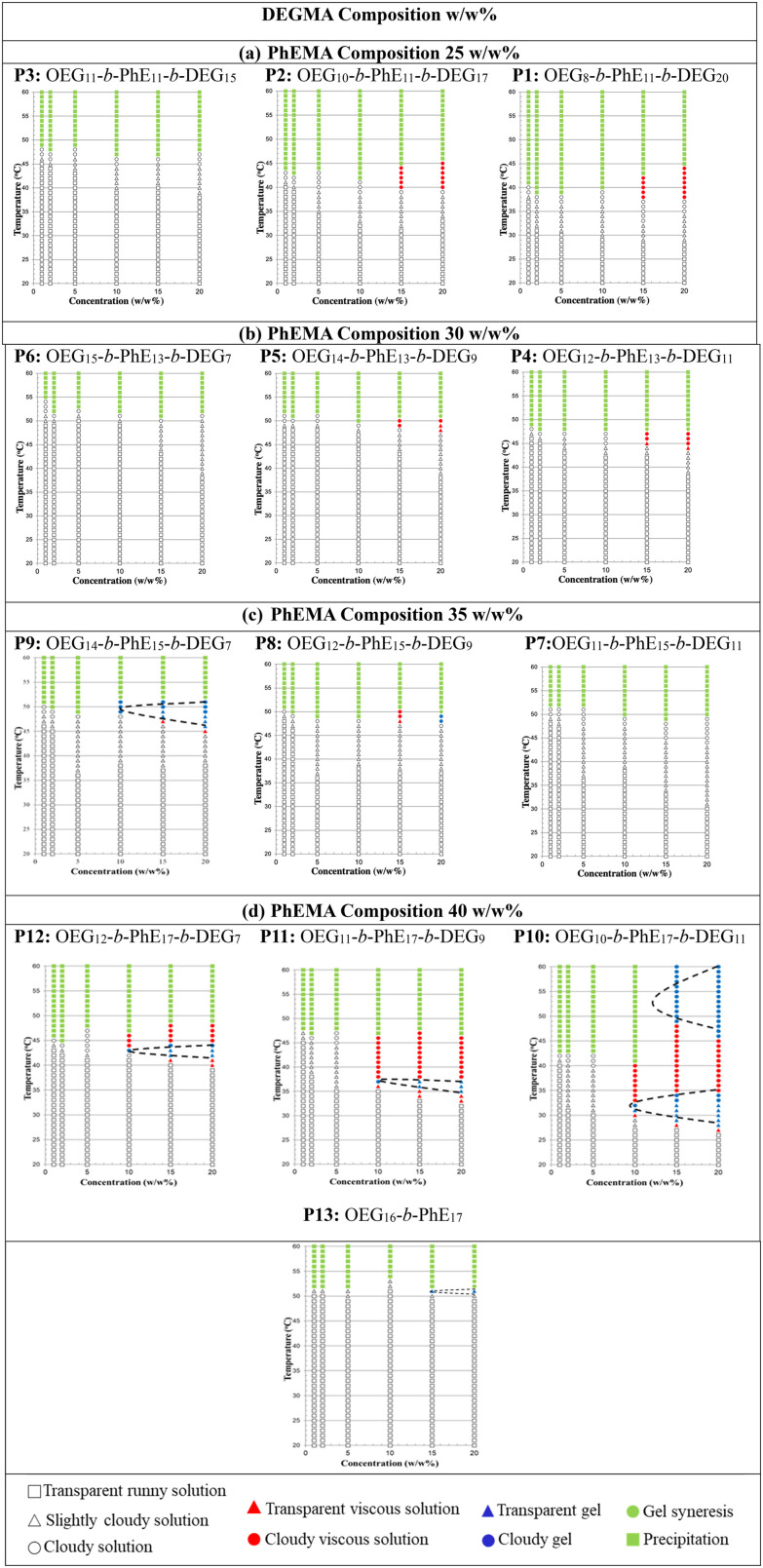
Phase diagrams of polymers P1–P13 OEGMA_*x*_-*b*-PhEMA_*y*_-*b*-DEGMA_*z*_ with different concentrations (1, 2, 5, 10, 15, 20 w/w%) in DI water. The composition of DEGMA increases from left to right and the composition of PhEMA increases from top to bottom. Four different phases can be observed: runny solution shown in the transparent shapes (square: clear solution, triangle: slightly cloudy solution and circle: cloudy solution), viscous solution shown in the red shapes (triangle: transparent viscous solution, circle: cloudy viscous solution), gel shown in the blue shapes (triangle: transparent gel, circle: cloudy gel) and two-phases shown in the green shapes (circle: gel syneresis, square: precipitation).

The gelation points of polymer P1–P13 OEGMA_*x*_-*b*-PhEMA_*y*_-*b*-DEGMA_*z*_ were investigated at different concentrations (1, 2, 5, 10, 15, 20 w/w%) in DI water by visual tests. The representative optical images illustrating the various states of the polymer solutions are shown in Fig. S5 (ESI†), and the phase diagrams of these polymers are presented in Fig. 7. The composition of OEGMA decreases from left to right and the composition of PhEMA increases from top to bottom. Four different phases can be observed: runny solution shown in the transparent shapes (square: clear solution, triangle: slightly cloudy solution and circle: cloudy solution), viscous solution shown in the red shapes (triangle: transparent viscous solution, circle: cloudy viscous solution), gel shown in the blue shapes (triangle: transparent gel, circle: cloudy gel) and two-phases shown in the green shapes (circle: gel syneresis, square: precipitation).

From all ABC triblock copolymers only five polymers with the higher PhEMA content (35 w/w% and 40 w/w%) were able to form thermogels (shown in blue triangles and circles). The need to have at least ∼35 w/w% hydrophobic content to form thermogels have been previously observed by our group in OEGMA based ABC triblock copolymers with different hydrophobic block.^33,36,47,51^ When the PhEMA content is 35 w/w%, P8 and P9 can form thermogels at a critical gelation concentration (CGC) of 20 and 10 w/w% copolymer in solution respectively. The gels are observed at 48 and 49 °C for P8 and P9 at the CGC, respectively. When the PhEMA content is 40 w/w%, all the copolymers can form gels. It is found that as the hydrophilicity of the copolymers increases, the gel region of the copolymers decreases, which means the copolymers formed gels at higher concentration and higher temperature, while P10 has the largest gel area, and P13 has the smallest one. This tendency is due to the inhibited hydrophobic effect. Moreover, it is observed that P10 has two gel regions, which may be due to possibly a change in the self-assembled polymer structure as the temperature increases. As the primary objective of this study was to identify the gelation temperature necessary for establishing optimal conditions for subsequent 3D printing, the underlying mechanisms of the two distinct gel regions were not investigated further. It was also observed, as expected, that when the concentration of the copolymer increases, the gelation temperature decreases, indicating that it can be tenably controlled by the polymer concentration. P10 is the best performing polymer that forms gels even at 31 °C at a concentration of 10 w/w%.

### Gelation points by rheology tests

**Fig. 8:**
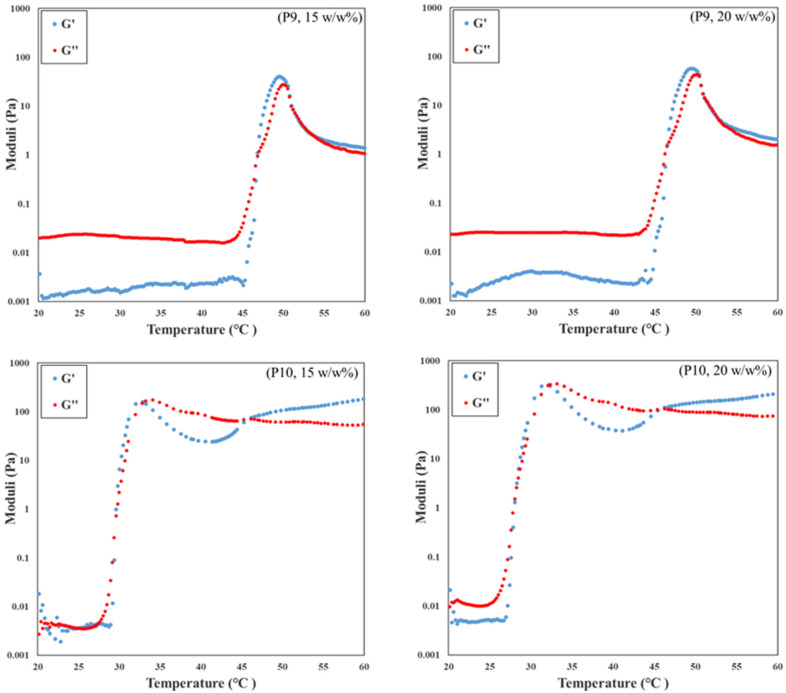
Changes of moduli (storage modulus *G*′ is denoted by blue circles and the loss modulus *G*′′ is denoted by red circles) with temperature for the polymer solutions of P9 and P10 that can form the gel at 15 w/w% and 20 w/w% in DI water.

The rheological properties of the samples that visually formed a thermogel were investigated over a temperature range of 20 °C to 60 °C. The results of P9 and P10 are shown in Fig. 8, while the rheological data for the other polymers are provided in Fig. S6 (ESI†). The storage modulus (*G*′) and loss modulus (*G*′′) are denoted in blue and red circles, respectively. In rheology the formation of a gel is defined as the point where the storage modulus exceeds the loss modulus, *i.e.*, when *G*′ > *G*′′.^36,57^ For all samples the gelation point agrees with the visual observations.

Specifically, for P9 and P10 at concentrations of 15 w/w% and 20 w/w%, there are two intersecting areas for each concentration. For P9, the gelation points at 15 w/w% and 20 w/w% are 46 °C and 45 °C, respectively, and they agree with the visual results within experimental error. The second crossovers of these two concentrations appear at 50 °C and 51 °C as the temperature increases. They also match with results obtained from the visual tests that both precipitations are observed at 53 °C. However, there is an interesting trend for P9 that the storage modulus still exceeds the loss modulus after the second crossover. It does not indicate the gel still exists because both magnitudes of two moduli decrease obviously compared with the maximum of two moduli. The magnitude of the modulus represents the strength and viscosity of the gel, and the phase separation usually happens when the magnitude decreases dramatically. It means there is a two-phase solution after the second crossover of both concentrations that is supported by visual observations. It is worth mentioning that the gel must exhibit sufficient strength to prevent flow upon tube inversion during the visual test. However, rheological test may still classify a very soft material as a gel, provided that the energy stored within the system exceeds the energy dissipated as heat. Thus, it is not surprising that the results of the rheological measurements do not perfectly align with those obtained by visual tests. In contrast, the storage modulus of P10 at the second intersecting area remains the same or a little higher than the storage modulus of the first intersecting area, which indicates that P10 can form gels twice. This phenomenon was also found in the visual test. Moreover, P10 has the maximum storage moduli among the tested copolymers, which means P10 forms the strongest gels and as expected the strength of the gel increased by increasing the polymer concentration.

### Application in 3D printing

**Fig. 9:**
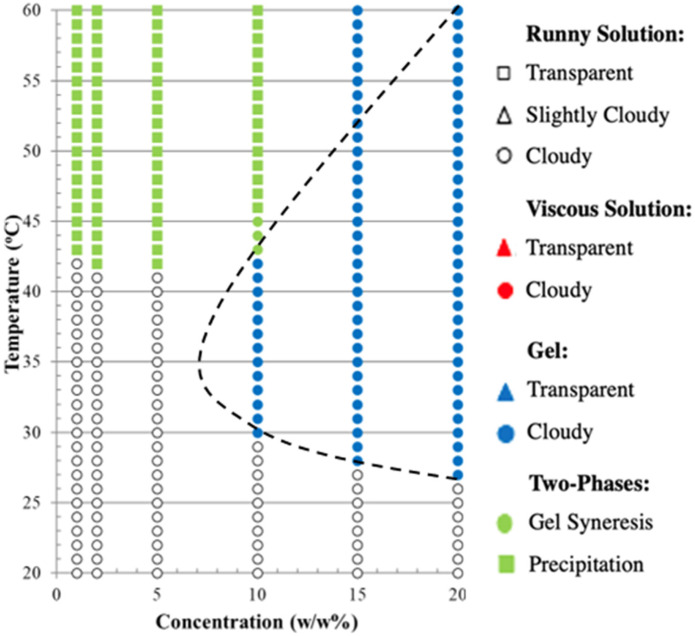
A phase diagram of polymers P10 OEGMA_10_-*b*-PhEMA_17_-*b*-DEGMA_11_ with different concentrations (1, 2, 5, 10, 15, 20 w/w%) mixed with 10 w/w% of graphene in DI water.

The most promising polymer, P10 OEGMA_10_-*b*-PhEMA_17_-*b*-DEGMA_11_ was chosen to be trialed as an “ink” for 3D printing based on the visual and rheological results. The hypothesis was that the benzyl ring in the PhEMA units will assist in stabilizing graphene. To identify what graphene/polymer ratio could be used to print, the phase diagram of P10 was constructed again in the presence of 10 w/w% graphene and the result is presented in Fig. 9. Representative optical images of the ink in various states as the temperature increases are provided in Fig. S7 (ESI†)

According to the Fig. 9, the gelation points of P10 at 10, 15 and 20 w/w% with 10 w/w% graphene are 30 °C, 28 °C and 27 °C respectively. When compared with the corresponding gelation points of P10 in Fig. 7, the gelation points in the presence of graphene are lower. Thus, it can be concluded that graphene has assisted the formation of a gel at lower temperatures, as was expected since it forms π–π interactions with the polymer. This observation was similar to a study where an oligolysine end functionalized Pluronic® F127 was mixed with graphene oxide (GO) and GO reduced the gelation point of Pluronic® F127 through electrostatic interactions.^58^

**Fig. 10:**
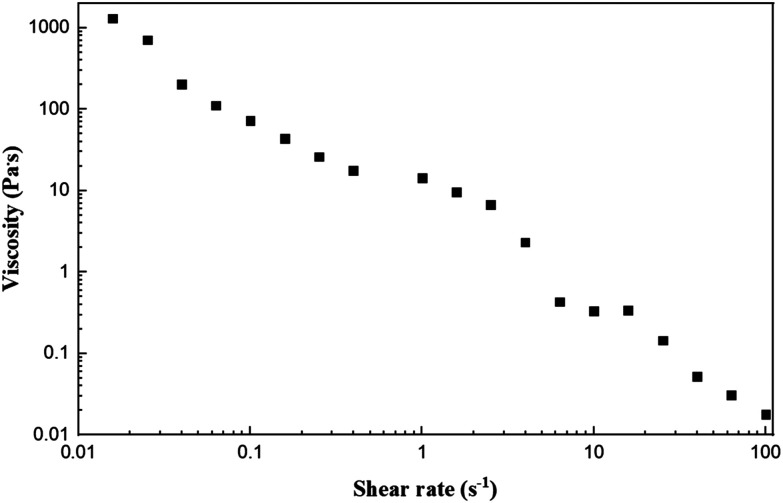
Viscosity-shear rate curve for the ink (10 w/w% P10 and 10 w/w% graphene in DI water) at 40 °C.

To minimize the copolymer content, a solution mixture containing 10 w/w% of P10 and 10 w/w% of graphene was selected as the ink. Initially, the shear-thinning behavior of the ink was examined at 40 °C, which is shown in Fig. 10. It can be seen from Fig. 10 that when the shear rate increases, the viscosity of the ink decreases significantly, indicating the expected shear-thinning property of the ink. This property enhances the extrudability of the ink through nozzles during the printing process, facilitating the fabrication of the desired printed structures.^6^

**Fig. 11:**
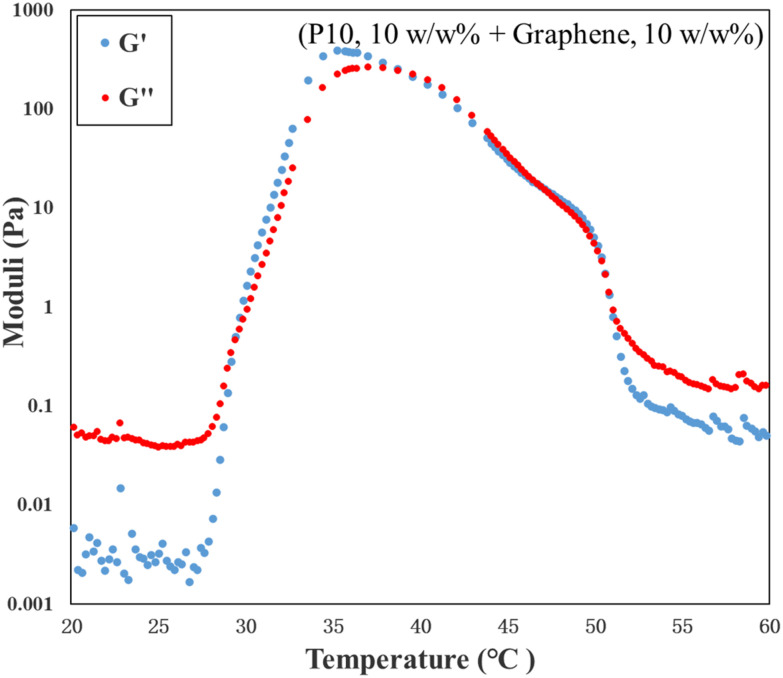
Changes of moduli (storage modulus *G*′ is denoted by blue circles and the loss modulus *G*′′ is denoted by red circles) with temperature for the ink (10 w/w% P10 and 10 w/w% graphene in DI water).

In addition, the rheological property of the ink has also been investigated by the rheology test. The result is shown in Fig. 11. From Fig. 11, it can be concluded that the ink formed gels at 30 °C and precipitated at 41 °C, which matches with the observations from the visual test.

Both the visual test observations and the rheological data of the ink support the conclusion that the interaction between hydrophobic graphene particles and the amphiphilic block copolymer significantly influences the self-assembly behavior and gelation properties of the system. The triblock terpolymer OEGMA_10_-*b*-PhEMA_17_-*b*-DEGMA_11_ self-assembles in aqueous solution due to its amphiphilic architecture: at lower temperatures, the hydrophilic OEGMA and DEGMA blocks form the corona, while the hydrophobic PhEMA block aggregates to form the micellar core. As temperature increases, the thermoresponsive DEGMA block becomes increasingly hydrophobic,^59^ contributing to micelle core formation and triggering gelation. Upon the addition of graphene, the polymer solution exhibits earlier gelation, a broader gelation temperature window, and an increased *G*′, indicating enhanced mechanical strength.

This enhancement is attributed to specific π–π stacking interactions between the benzene rings of the PhEMA block and the π-conjugated graphene surface. These non-covalent interactions drive graphene to preferentially localize in the hydrophobic micelle core during self-assembly. Rather than disrupting micelle formation, the graphene particles integrate into the micellar structures, reinforcing the micellar network and enhancing intermicellar connectivity. In this system, graphene acts as a physical crosslinking or reinforcing agent within the gel. This phenomenon is consistent with previous reports showing that graphene or graphene oxide, when incorporated into amphiphilic polymer systems, can improve viscoelastic properties and gel stability *via* physical entanglement and π–π interactions.^60–62^

Therefore, an aqueous solution of 10 w/w% P10 and 10 w/w% graphene was selected as the ink because it presents the highest graphene/polymer ratio, and it is still able to form a thermogel. The polymer/graphene ink was prepared and processed at ambient temperature, followed by being printed using a 3D printer onto a heated substrate maintained at 40 °C to achieve stable serpentine structures. After printing, the polymer-based structures were air-dried overnight to remove any residual solvent. The dried samples were then annealed in a tubular furnace at 350 °C for 30 minutes to promote the carbonisation of the terpolymer,^63,64^ ensuring proper stabilisation of the printed structures prior to the SEM and four-point probe conductivity tests.

**Fig. 12:**
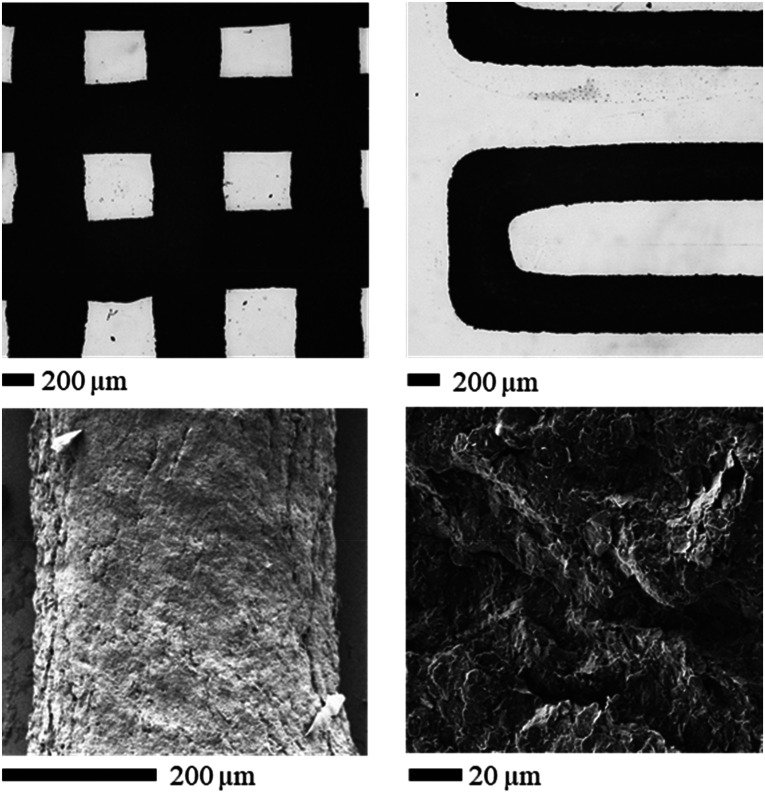
SEM images of the printed structures based on 10 w/w% of P10 OEGMA_10_-*b*-PhEMA_17_-*b*-DEGMA_11_ with 10 w/w% of graphene. (a) A two-layer cross-grid pattern, (b) a single-layer serpentine pattern, (c and d) surface morphology of the printed structure.

**Fig. 13:**
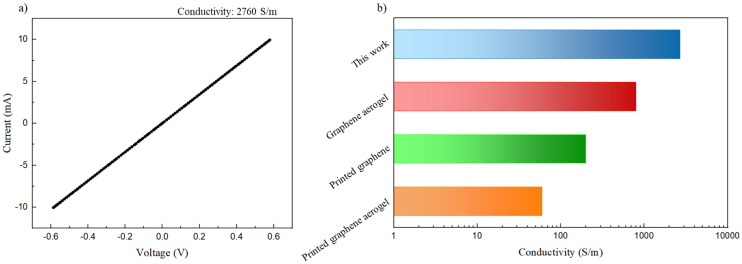
(a) *I*–*V* curve of the printed structure; (b) electrical conductivity of the printed structure compared with three different DIW inks.^65–67^

The successfully printed structure is shown in Fig. 12. Fig. 12 shows that the edges of the printed structure are well defined, which indicates the high potential of this kind of ink. The electrical conductivity of this printed structure was tested *via* a four-electrode measurement (Fig. 13a), exhibiting an electrical conductivity of ∼2760 S m^−1^. This conductivity exceeds that of recently reported graphene structures fabricated using Direct Ink Writing (Fig. 13b),^65–67^ attributed to the utilization of graphene nanoplatelets instead of reduced graphene oxide and the minimal requirement of polymeric binder during the printing procedure. This kind of structure might find application as sensors and energy storage systems owing to the conductive and capacitive nature of graphene platelets.^63^ This shows that with further studies and optimization this type of ABC triblock copolymer could be used to print graphene containing inks.

It is important to note that this study focused on a single graphene addition (10 w/w%), and this study did not systematically investigate the effect of graphene concentration. However, previous studies have observed that polymer-graphene systems can exhibit concentration-sensitive rheological and structural properties, where excessive graphene content may lead to micelle disruption or phase separation.^61,68^ Future work may focus on optimizing the graphene ratio to further tailor gelation behavior and mechanical properties. Despite this limitation, this study clearly demonstrates that introducing 10 w/w% graphene enhances the thermoresponsive gelation and mechanical performance of the triblock terpolymer system and this type of ABC triblock copolymer could be used to print graphene containing inks.

## Conclusions

In this study, a new series of ABC triblock copolymers and two diblock copolymers (AB, CB) were successfully synthesized *via* GTP based on OEGMA (A), PhEMA (B) and DEGMA (C). The targeted MMs for all polymers were the same but thirteen polymers of varying composition were fabricated and their thermoresponsive and aqueous solution properties were investigated. These properties were strongly affected by the composition of the ABC triblock terpolymers and five out of thirteen polymers were found to form thermogels at elevated temperatures. The most promising thermogel, P10 OEGMA_10_-*b*-PhEMA_17_-*b*-DEGMA_11_, was then mixed with (10 w/w%) graphene and the gelation temperature was decreased as graphene assisted with the formation of the physical gel. Finally, a 10 w/w% graphene and 10 w/w% OEGMA_10_-*b*-PhEMA_17_-*b*-DEGMA_11_ “ink” was used to successfully print a stable structure, which has an excellent electrical conductivity of ∼2760 S m^−1^.

## Author contributions

BF and XL carried most of the polymer synthesis and characterization and wrote the first draft. ST did the printing of graphene inks. APC helped with polymer synthesis, data analysis and the cloud point temperature determination. AEP assisted with the supervision of the project. CM supervised the graphene ink printing. TKG had the idea, supervised, coordinated the project and edited the manuscript.

## Abbreviations

CP: Cloud point
DEGMA: Di(ethylene glycol) methyl ether methacrylate (DEGMA)
DLS: Dynamic light scattering
SEC: Gel permeation chromatography
GTP: Group transfer polymerization
MTS: Methyltriemthylsilyl dimethylketene acetal
MM: Molar mass
NMR: Nuclear magnetic resonance
OEGMA: Oligo(ethylene glycol) methyl ether methacrylate
PhEMA: 2-Phenylethyl methacrylate
PTFE: Poly(tetrafluoroethylene)
TBABB: Tetrabutylammonium bibenzoate
THF: Tetrahydrofuran.

## Conflicts of interest

There are no conflicts to declare.

## Supplementary Material

LP-003-D5LP00071H-s001

## Data Availability

The data supporting this article has been included in the ESI of this manuscript.†
