# Supplementary material for: Graphene inks for printing based on thermoresponsive ABC triblock terpolymer gels
Source: RSC Appl Polym. 2025 Jun 2;3(4):973–89. doi: 10.1039/d5lp00071h (PMC12142586; doi:10.1039/d5lp00071h)
Supplement: LP-003-D5LP00071H-s001 [file LP-003-D5LP00071H-s001.pdf]

Supplementary Information (SI) for RSC Applied Polymers.
This journal is © The Royal Society of Chemistry 2025

Supporting Information

Graphene Inks for Printing based on Thermoresponsive ABC Triblock Terpolymers Gels

*Xu Liu[#], Bailin Feng,[#] Stefano Tagliaferri, Anna P. Constantinou, Alexandra E. Porter, Cecilia Mattevi, Theoni K. Georgiou**

Department of Materials, Imperial College London, Royal School of Mines, Exhibition Road, SW7 2AZ, London, United Kingdom

#: These authors contributed to the work equally and should be regarded as co-first authors.

P2)

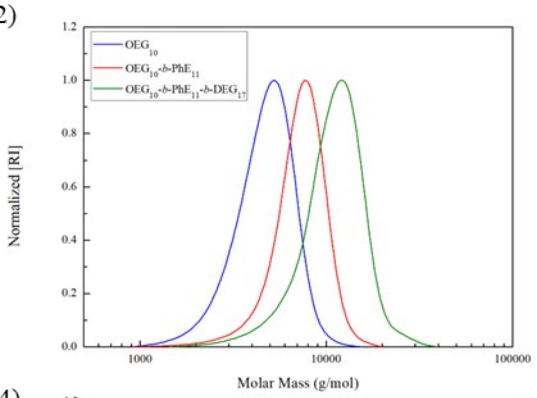

P3)

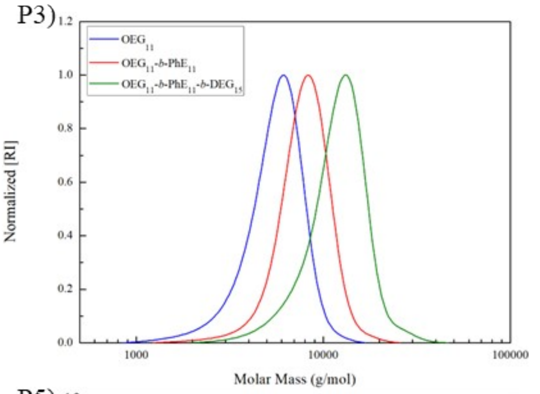

P4)

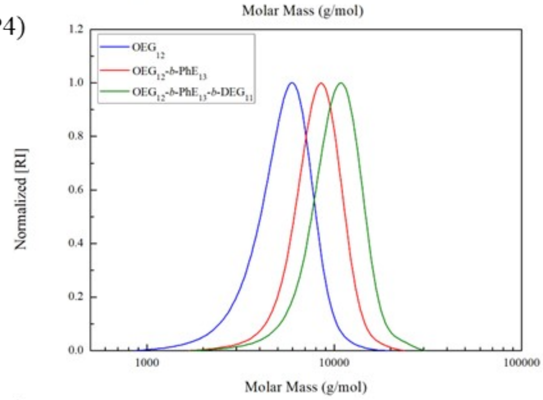

P5)

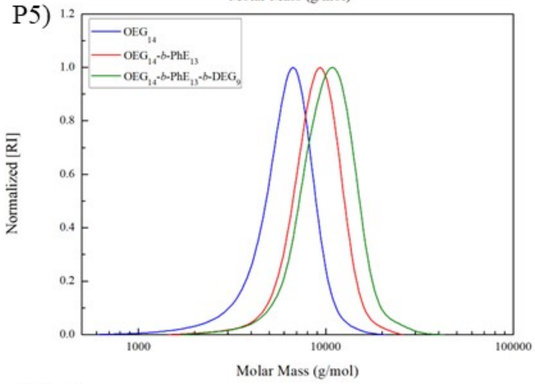

P6)

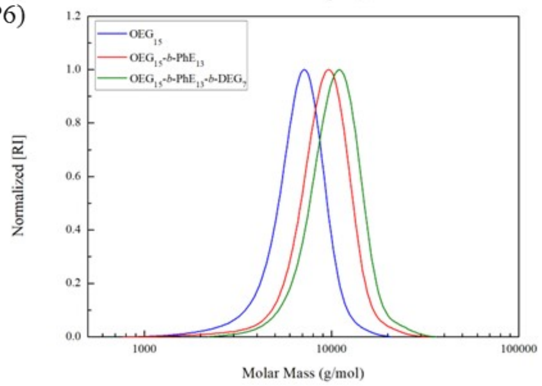

P7)

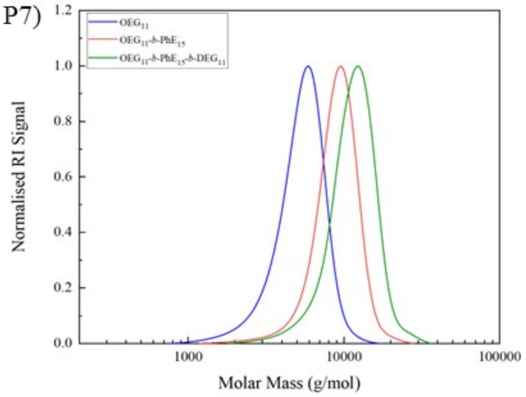

P8)

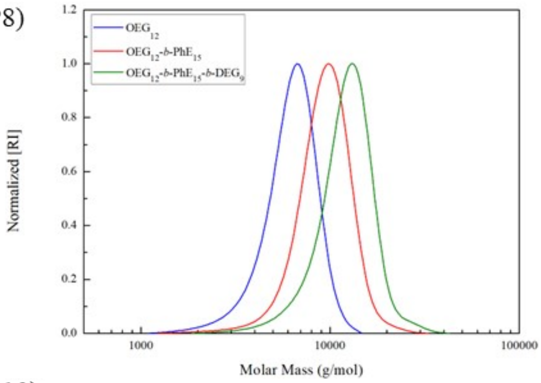

P9)

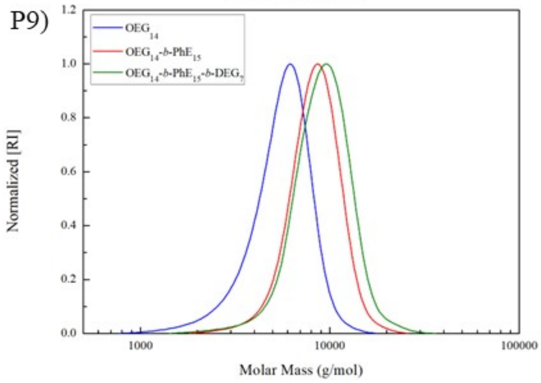

P10)

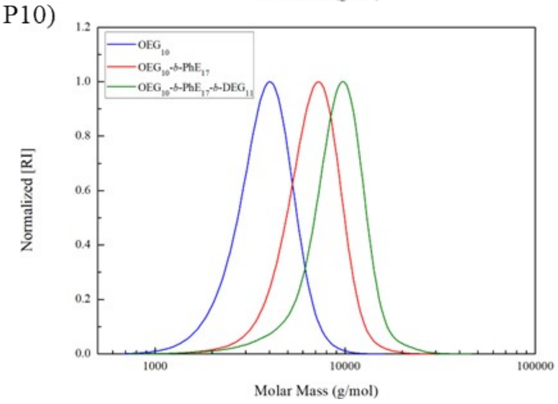

P11)

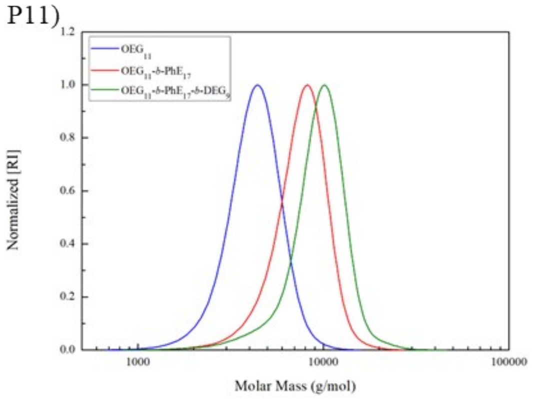

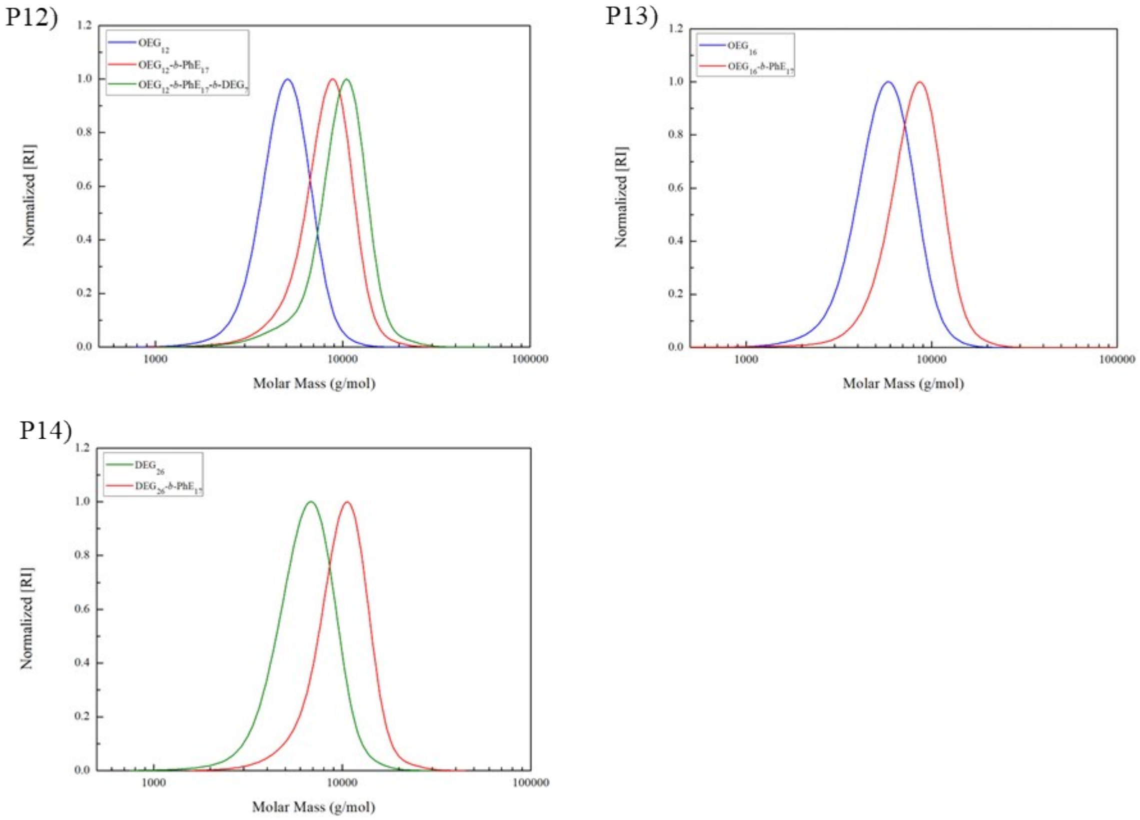

Figure S1. The GPC curves of $OEGMA_x$-*b*-$PhEMA_y$-*b*-$DEGMA_z$ was shown in the green curve and the precursors $OEGMA_x$ and $OEGMA_x$-*b*-$PhEMA_y$ were denoted with blue and red curves respectively.

Table S1: Theoretical polymer structure, target, and experimental molecular mass (M_n), dispersity ($Đ$), theoretical and experimental composition of polymers.

No.	Theoretical Polymer Structure [a]	Target MM (g/mol)	M_n [b] (g/mol)	$Đ$ [b]	w/w% OEG-*b*-PhE-*b*-DEG	
					Theoretical	^{1}H NMR
**P1**	OEG_8	2460	4060	1.14	100-0-0	100-0-0
	OEG_8-*b*-PhE_{11}	4510	6420	1.12	54-46-0	56-44-0
	OEG_8-*b*-PhE_{11}-*b*-DEG_{20}	8200	9700	1.20	30-25-45	31-24-45
**P2**	OEG_{10}	2870	4320	1.14	100-0-0	100-0-0
	OEG_{10}-*b*-PhE_{11}	4920	6730	1.12	58-42-0	60-40-0
	OEG_{10}-*b*-PhE_{11}-*b*-DEG_{17}	8200	9870	1.17	35-25-40	38-25-37
**P3**	OEG_{11}	3280	5080	1.14	100-0-0	100-0-0
	OEG_{11}-*b*-PhE_{11}	5330	7420	1.11	61-39-0	64-36-0
	OEG_{11}-*b*-PhE_{11}-*b*-DEG_{15}	8200	10100	1.15	40-25-35	43-24-33
**P4**	OEG_{12}	3690	4910	1.15	100-0-0	100-0-0
	OEG_{12}-*b*-PhE_{13}	6150	7680	1.10	60-40-0	62-38-0
	OEG_{12}-*b*-PhE_{13}-*b*-DEG_{11}	8200	9550	1.12	45-30-25	47-29-24
**P5**	OEG_{14}	4100	5570	1.15	100-0-0	100-0-0
	OEG_{14}-*b*-PhE_{13}	6560	8390	1.10	63-37-0	64-36-0
	OEG_{14}-*b*-PhE_{13}-*b*-DEG_9	8200	9550	1.13	50-30-20	52-30-18
**P6**	OEG_{15}	4510	6200	1.13	100-0-0	100-0-0
	OEG_{15}-*b*-PhE_{13}	6970	8470	1.13	65-35-0	68-32-0
	OEG_{15}-*b*-PhE_{13}-*b*-DEG_7	8200	9810	1.11	55-30-15	59-28-13
**P7**	OEG_{11}	3280	4850	1.15	100-0-0	100-0-0
	OEG_{11}-*b*-PhE_{15}	6150	8360	1.12	53-47-0	56-44-0
	OEG_{11}-*b*-PhE_{15}-*b*-DEG_{11}	8200	10500	1.14	40-35-25	43-33-24
**P8**	OEG_{12}	3690	5780	1.11	100-0-0	100-0-0
	OEG_{12}-*b*-PhE_{15}	6560	8720	1.12	56-44-0	59-41-0
	OEG_{12}-*b*-PhE_{15}-*b*-DEG_9	8200	11300	1.13	45-35-20	47-33-20
**P9**	OEG_{14}	4100	5060	1.15	100-0-0	100-0-0
	OEG_{14}-*b*-PhE_{15}	6970	7880	1.10	59-41-0	59-41-0
	OEG_{14}-*b*-PhE_{15}-*b*-DEG_7	8200	8490	1.13	50-35-15	51-34-15
**P10**	OEG_{10}	2870	3400	1.14	100-0-0	100-0-0
	OEG_{10}-*b*-PhE_{17}	6150	6200	1.13	47-53-0	48-52-0
	OEG_{10}-*b*-PhE_{17}-*b*-DEG_{11}	8200	8200	1.16	35-40-25	36-39-25
**P11**	OEG_{11}	3280	3900	1.10	100-0-0	100-0-0
	OEG_{11}-*b*-PhE_{17}	6560	7000	1.12	50-50-0	52-48-0
	OEG_{11}-*b*-PhE_{17}-*b*-DEG_9	8200	8600	1.15	40-40-20	42-39-19
**P12**	OEG_{12}	3690	4600	1.11	100-0-0	100-0-0

	OEG_{12}-*b*-PhE_{17}	6970	7600	1.13	53-47-0	55-45-0
	OEG_{12}-*b*-PhE_{17}-*b*-DEG_7	8200	9000	1.14	45-40-15	47-38-15
**P13**	OEG_{16}	4920	5100	1.15	100-0-0	100-0-0
	OEG_{16}-*b*-PhE_{17}	8200	8300	1.17	60-40-0	61-39-0
**P14**	DEG_{26}	4920	6700	1.15	0-0-100	0-0-100
	DEG_{26}-*b*-PhE_{17}	8200	10400	1.17	0-40-60	0-40-60

[a] OEG, PhE and DEG are further abbreviations of oligo(ethylene glycol) methyl ether methacrylate, 2-phenylethyl methacrylate and di(ethylene glycol) methyl ether methacrylate, respectively.

[b] Number-average molar mass (M_n) and dispersity ($Đ$) were determined by SEC. The SEC was calibrated by using poly(methyl methacrylate) (PMMA) standard samples.

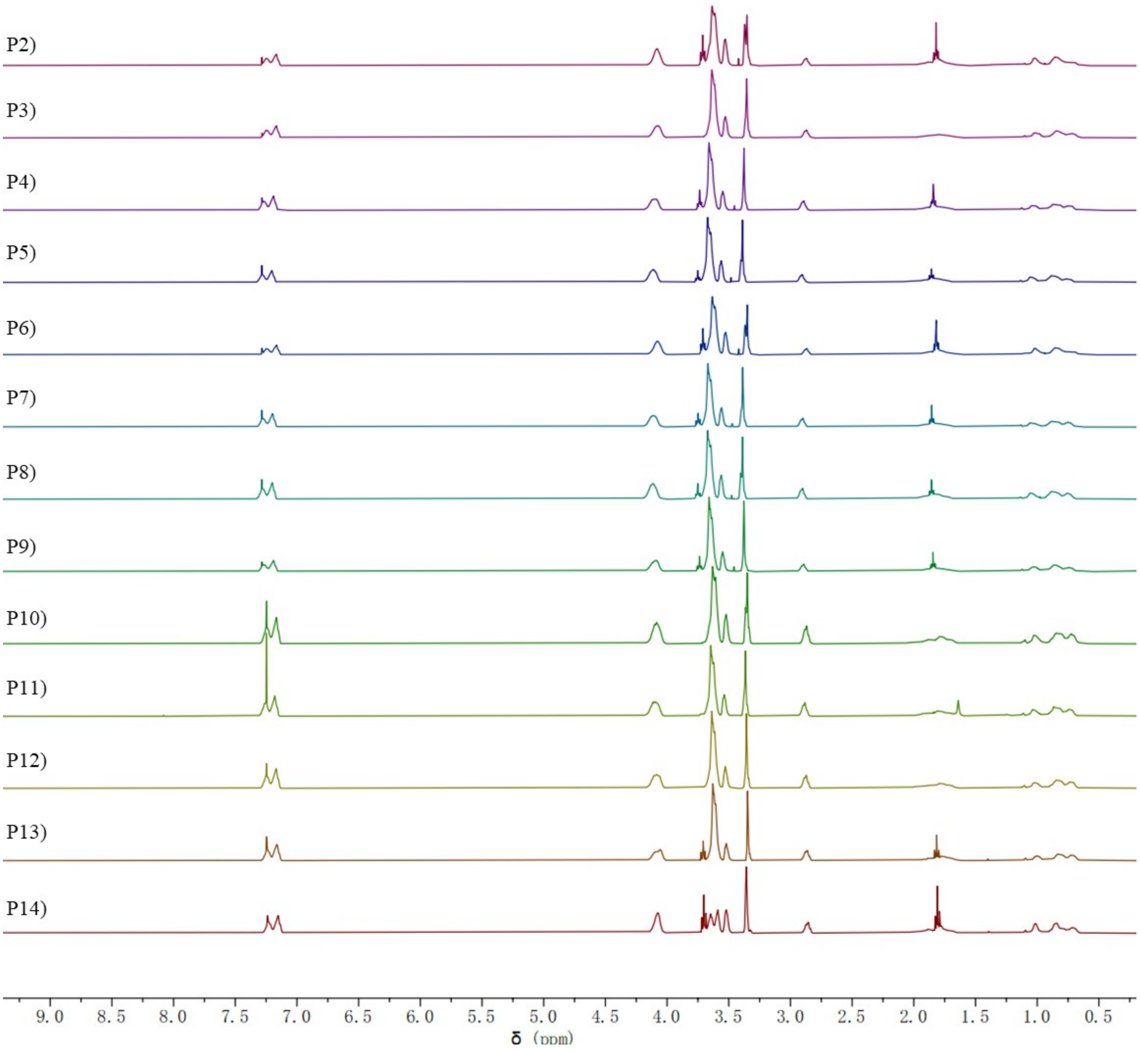

Figure S2: ^{1}H NMR spectra of the triblock polymer $OEGMA_x$-*b*-$PhEMA_y$-*b*-$DEGMA_z$ in the order of P2 to P14 from top to bottom.

P1)

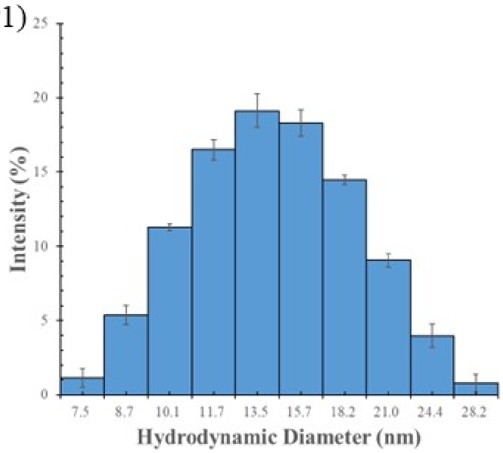

P2)

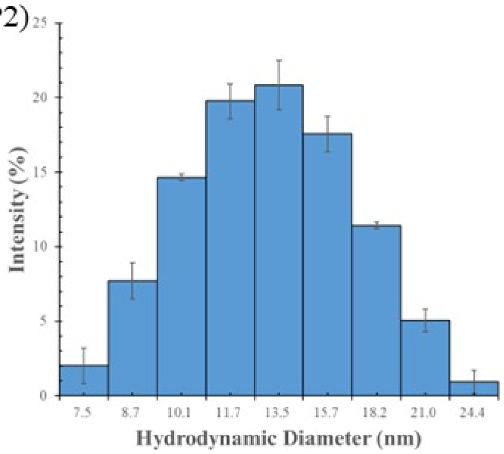

P3)

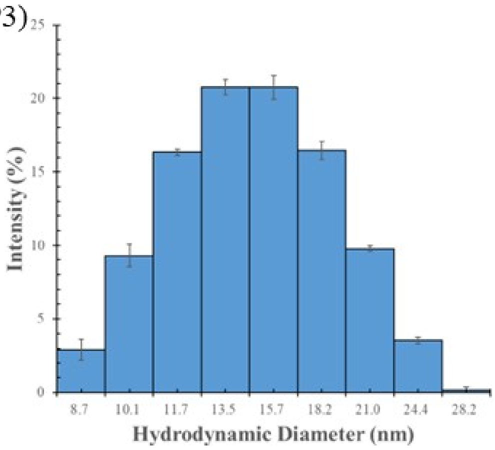

P4)

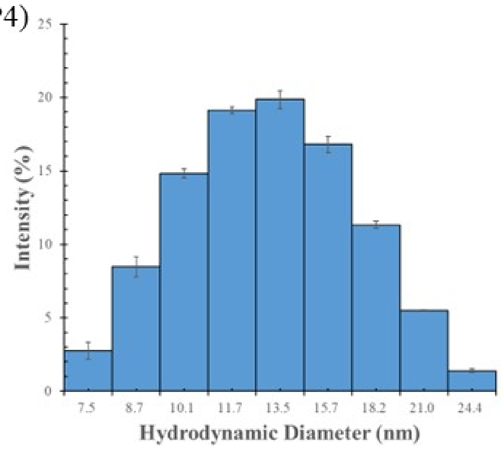

P5)

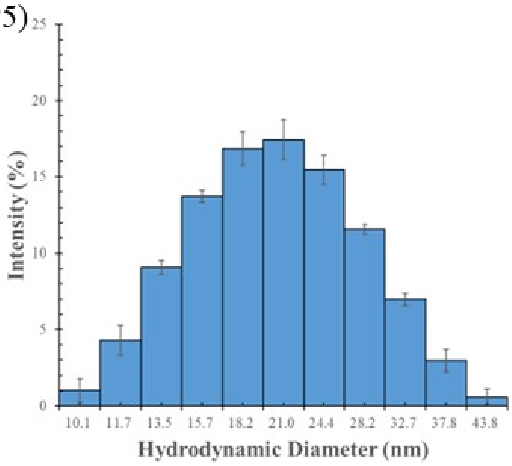

P6)

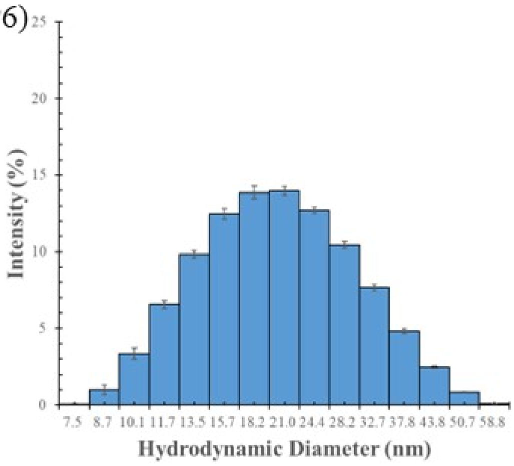

P7)

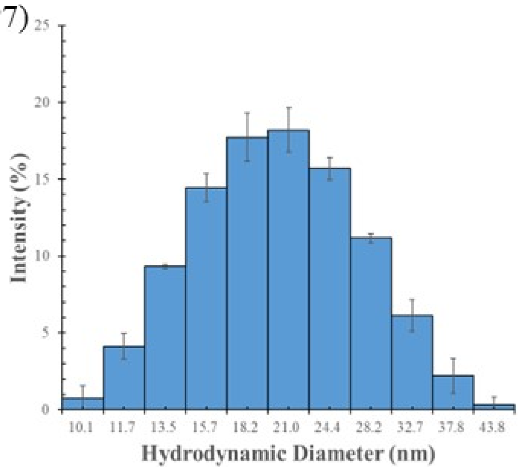

P8)

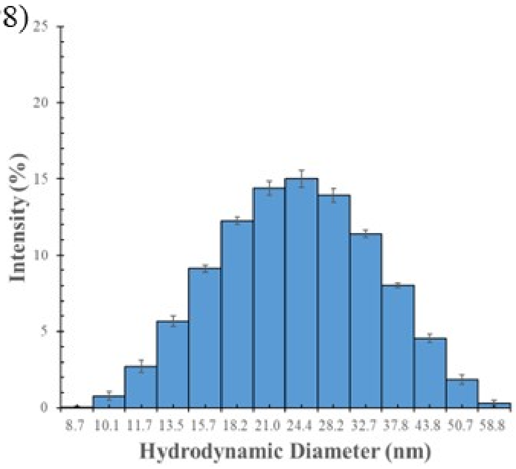

P9)

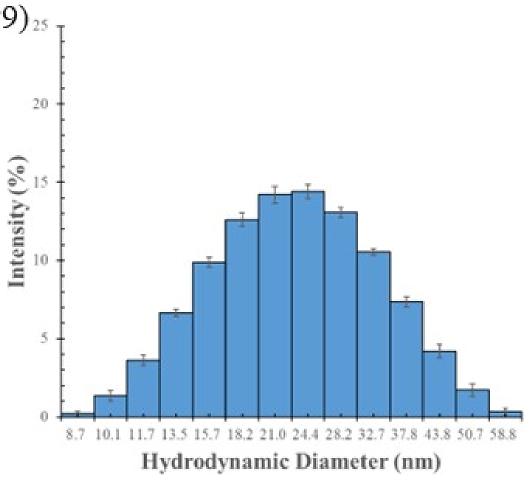

P10)

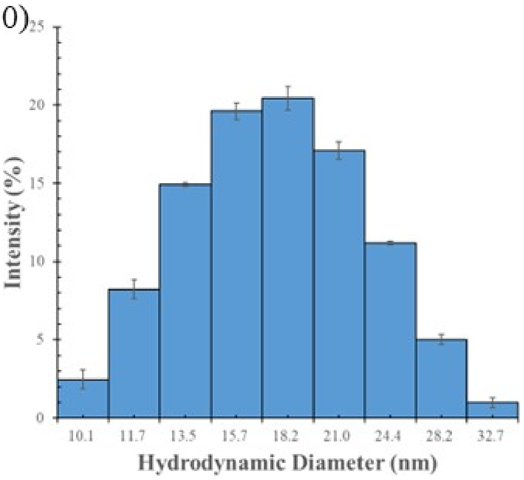

P11)

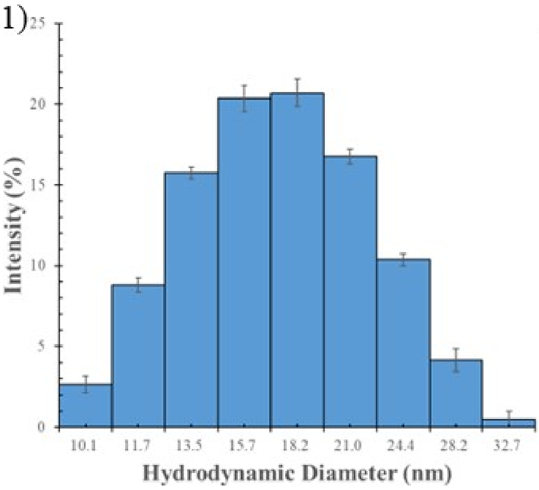

P12)

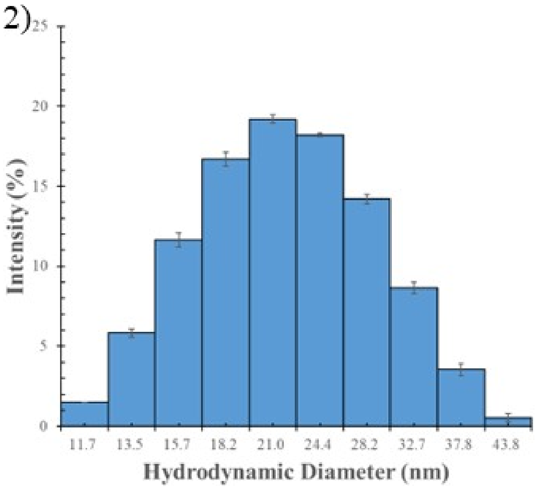

P13)

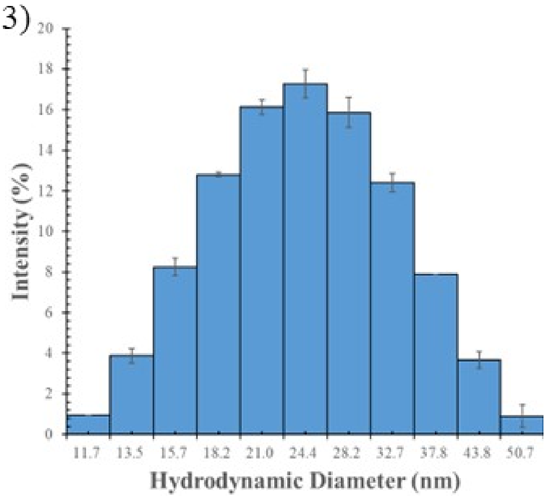

Figure S3. Histograms of DLS by intensity of the triblock polymers solutions in DI water at 1 w/w%.

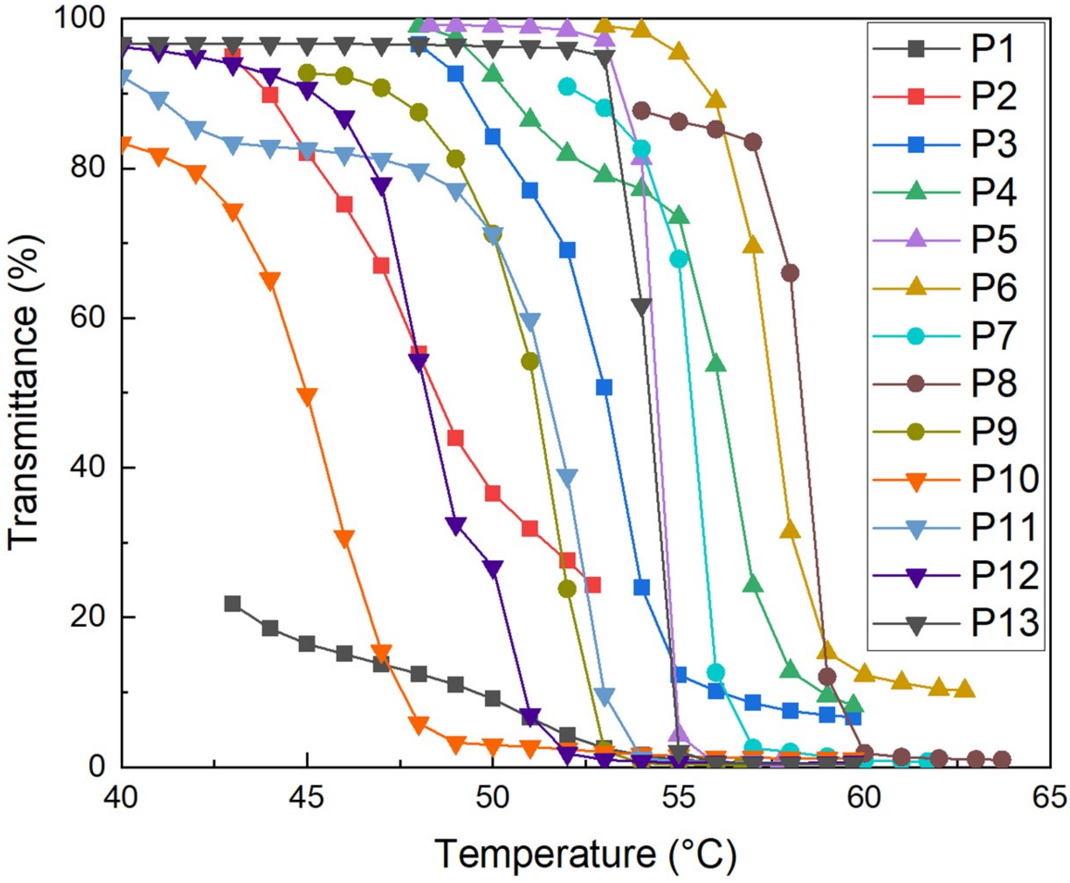

Figure S4. Transmittance as a function of temperature, obtained by the thermal mode on 1 w/w% solution in DI water. Different polymers are represented by different

colours.

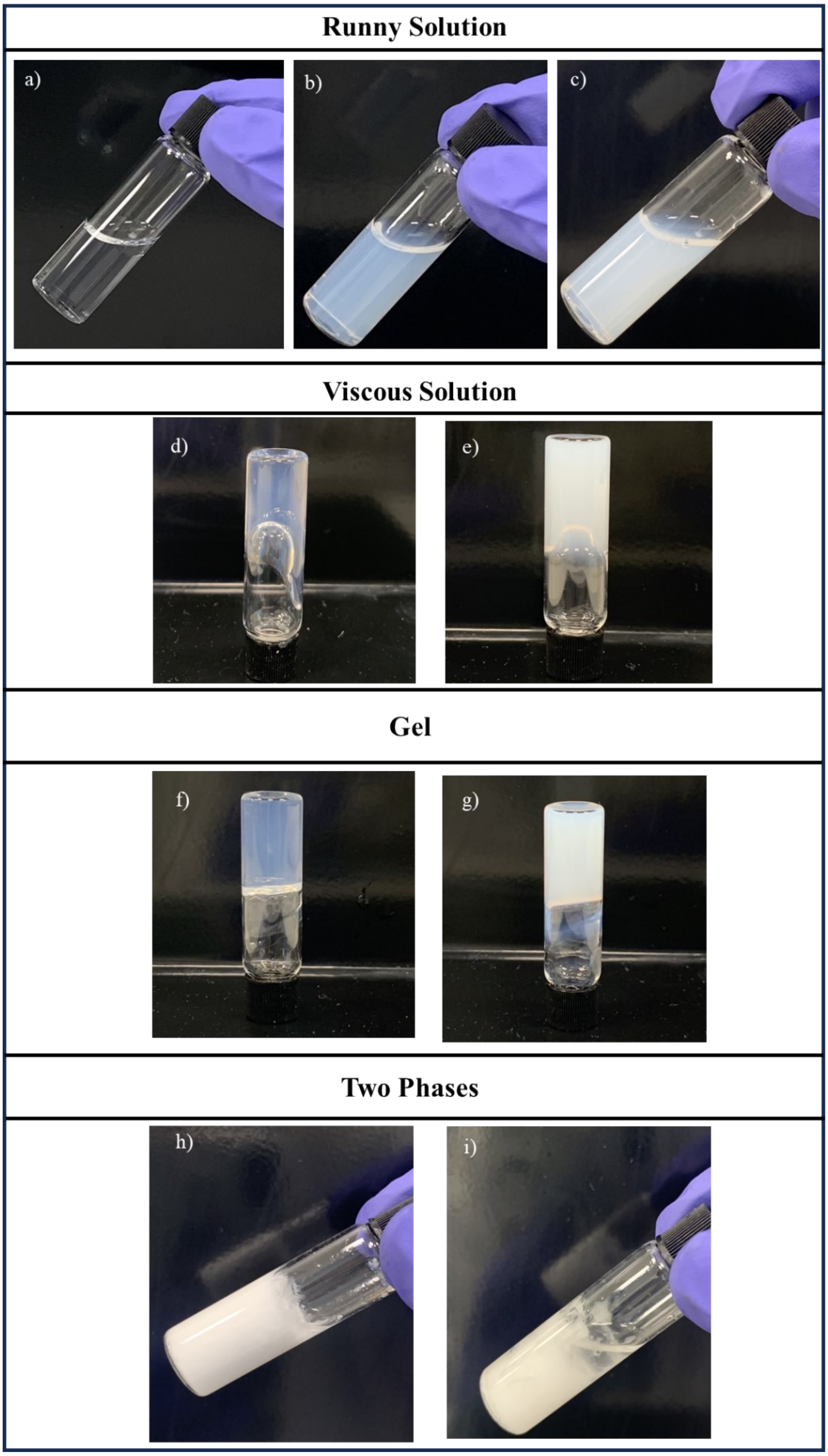

Figure S5. Representative optical images illustrating the various states of the polymer solution as observed through visual inspection: a) clear solution, b) slightly cloudy solution, c) cloudy solution, d) transparent viscous solution, e) cloudy viscous solution, f) transparent gel, g) cloudy gel, h) gel syneresis, and i) precipitation.

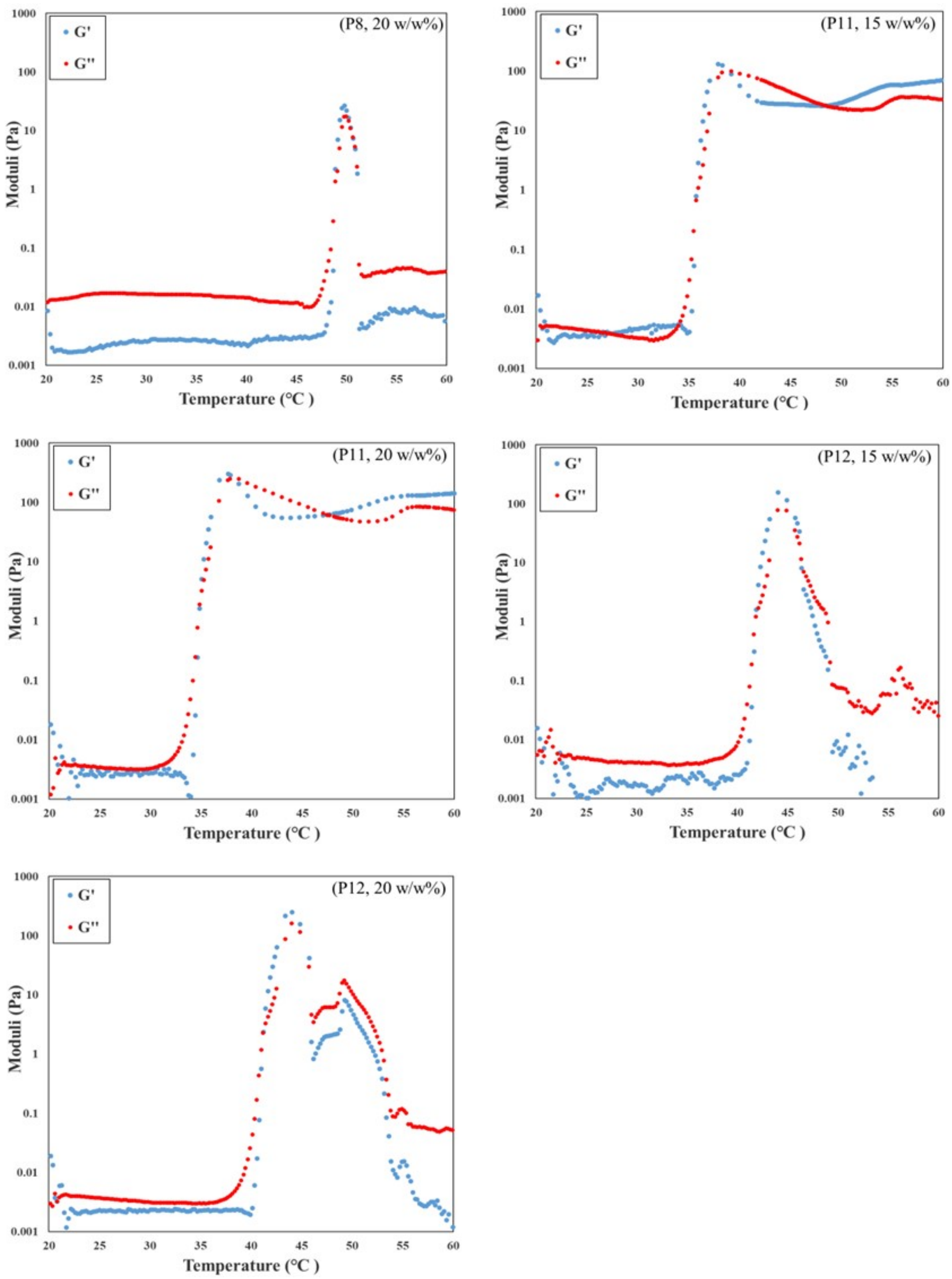

Figure S6. Temperature-dependent changes in the storage modulus (G′, blue circles) and loss modulus (G″, red circles) for polymer solutions of P8, P11 and P12 at concentrations of 15 wt% and 20 wt% in deionized (DI) water, respectively. All the polymers exhibited thermogelation behavior under the tested conditions.

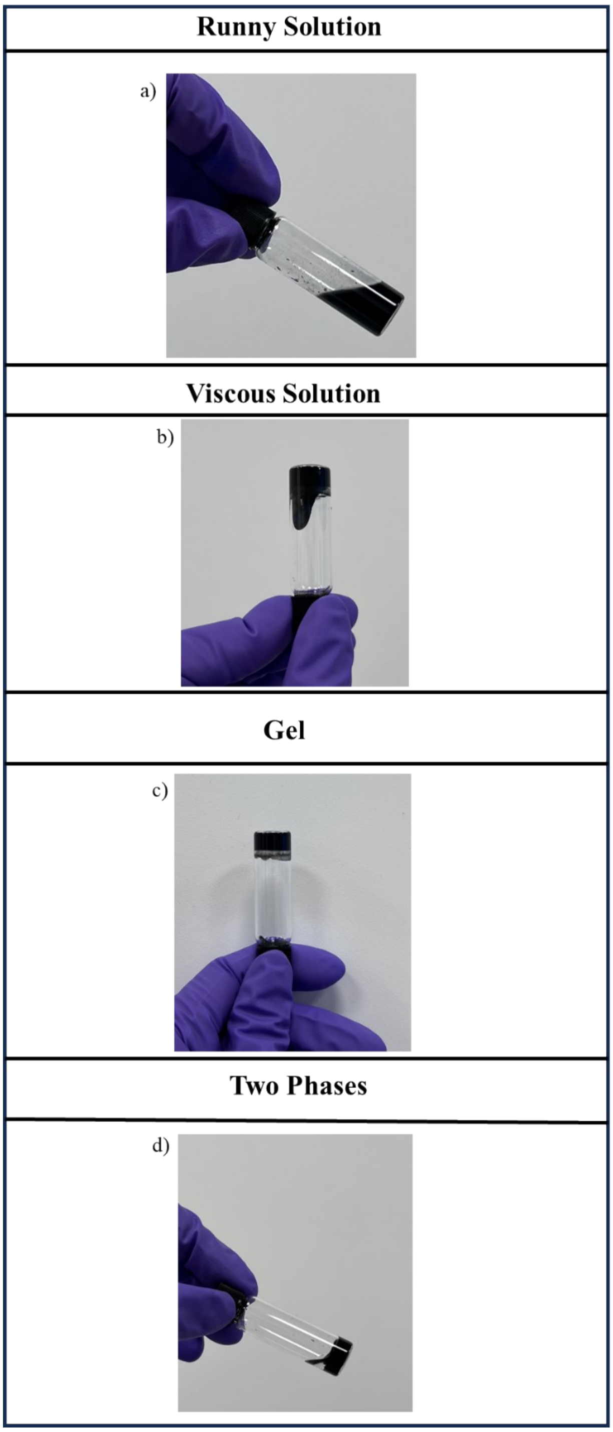

Figure S7. Representative optical images illustrating the various states of the polymer/graphene solution as observed through visual inspection: a) cloudy solution, b) cloudy viscous solution, c) cloudy gel, d) precipitation
